# Supplementary material for: Trends in alcohol-related injury admissions in adolescents in Western Australia and England: population-based cohort study
Source: BMJ Open. 2017 May 29;7(5):e014913. doi: 10.1136/bmjopen-2016-014913 (PMC5623454; doi:10.1136/bmjopen-2016-014913)
Supplement: Author's manuscript [file bmjopen-2016-014913.draft_revisions.pdf]

Trends in injury admissions associated with alcohol in adolescents in Western Australia and England

Journal:	*BMJ Open*
Manuscript ID	bmjopen-2016-014913
Article Type:	Research
Date Submitted by the Author:	01-Nov-2016
Complete List of Authors:	O'Donnell, Melissa; University of Western Australia, Telethon Kids Institute<br>Sims, Scott; Telethon Kids Institute, University of Wester Australia,<br>Maclean, Miriam; Telethon Kids Institute, University of Western Australia,<br>Gonzalez-Izquierdo, Arturo; University College London<br>Gilbert, Ruth; University College London, Institute of Child Health<br>Stanley, Fiona; University of Western Australia, Telethon Kids Institute
**Primary Subject Heading**:	Public health
Secondary Subject Heading:	Epidemiology, Public health
Keywords:	Non-accidental injury < PAEDIATRICS, alcohol, youth, adolescent, trends, injury

SCHOLARONE™ Manuscripts

For peer review only - http://bmjopen.bmj.com/site/about/guidelines.xhtml

Running Head: TRENDS IN ALCOHOL-RELATED INJURY ADMISSIONS

Title: Trends in injury admissions associated with alcohol in adolescents in Western Australia and England

Melissa O'Donnell, BPsych (Hons), MPsych, GradDip Ed, PhD, NHMRC Postdoctoral Research Fellow, Telethon Kids Institute, University of Western Australia, Perth, Australia. Email: melissao@ichr.uwa.edu.au

Scott Sims, BSc, MBiostat, Data Analyst, Telethon Kids Institute, University of Western Australia, Perth, Australia. Email: Scott.Sims@telethonkids.org.au

Miriam J Maclean, BPsych (Hons), MSc, Telethon Kids Institute, University of Western Australia, Perth, Australia. Email: Miriam.Maclean@telethonkids.org.au

Arturo Gonzalez-Izquierdo, PhD, Data Scientist, Farr Institute of Health Informatics Research, University College London, London. Email: arturo.gonzalez-izquierdo@ucl.ac.uk

Ruth Gilbert, MD, MSc, Professor of Clinical Epidemiology, Population Policy and Practice Programme, University College London, Institute of Child Health, London. Email: r.gilbert@ucl.ac.uk

For peer review only

For peer review only - http://bmjopen.bmj.com/site/about/guidelines.xhtml

Fiona J Stanley, MD, MSc, FFPHM, FAFPHM, Research Professor, Telethon Kids Institute, University of Western Australia, Perth, Australia. Email: Fiona.Stanley@telethonkids.org.au

Corresponding Author and Address: Melissa O'Donnell, Telethon Kids Institute, University of Western Australia, 100 Roberts Road, Subiaco, Western Australia. Telephone: (08) 9489 7778. Email: Melissa.O'Donnell@telethonkids.org.au

Keywords: Injury rates, trends, alcohol, adolescents, youth

Number of Tables=1

Number of Figures=3

Word Count=2921

For peer review only

For peer review only - http://bmjopen.bmj.com/site/about/guidelines.xhtml

What is already known on this subject?

Alcohol-related harm in adolescents has become a global health priority causing a large burden on hospitals for injury admissions caused by both accidents and violence. We have previously shown that that violence-related injury accounts for almost a quarter of all injury admissions to hospital for adolescents aged 16 to 17 years. Further information is required on the trends in alcohol-related admissions in different groups and the causes of injuries to guide further prevention and early intervention efforts.

What this study adds?

England has seen a decline in alcohol-related injury admissions for adolescents aged 13-17 years since 2007, while Western Australia has seen a significant increase. In England self-harm is the most frequently recorded cause of alcohol-related injury compared to Western Australia where unintentional injury is most common with violence-related harm increasing for boys and girls. Interventions to reduce the affordability and availability of alcohol are effective in reducing harm with England's rise in alcohol taxation a possible reason for the reduction in harm.

For peer review only - http://bmjopen.bmj.com/site/about/guidelines.xhtml

Abstract

Background: Alcohol-related harm in young people is now a global health priority. We examined trends in hospital admissions for alcohol-related injuries for adolescents in Western Australia (WA) and in England, identified groups most at risk and determined causes of injuries.

Methods: Annual incidence rates for alcohol-related injury rates were calculated using population-level hospital admissions data for WA and England. We compared trends in different types of alcohol-related injury by age and gender.

Results: Despite a decrease in the overall rate of injury admissions for 13-17 year olds in WA, alcohol-related injuries have increased significantly from 1990-2009 (from 8 to 12 per 10,000). Conversely, alcohol-related injury rates have declined in England since 2007. In England self-harm is the most frequently recorded cause of alcohol-related injury. In WA, unintentional injury is most common, however violence-related harm is increasing for boys and girls.

Conclusions: Alcohol-related harm of sufficient severity to require hospital admission is increasing among adolescents in Western Australia. Declining trends in England suggests this trend is not inevitable or irreversible. More needs to be done to address alcohol-related harm, and ongoing monitoring is required to assess the effectiveness of strategies.

Strengths and limitations of this study

- This study investigates trends in adolescent hospital admissions for injuries and the documented causes of these injuries.
- While trends in alcohol related admissions has been reported in previous research this adds to the knowledge of trends in different age groups and causes of injury.

For peer review only - http://bmjopen.bmj.com/site/about/guidelines.xhtml

- The limitations of this research is that only individuals admitted for injuries who have an alcohol-related diagnosis will be captured not when the perpetrator had been affected by alcohol therefore underestimating rates of injury associated with alcohol use.
- There also could be changes in coding practices which may contribute to changes in trends.

For peer review only

For peer review only - http://bmjopen.bmj.com/site/about/guidelines.xhtml

INTRODUCTION

Substance use in young people is considered a global health priority with young people highlighted as a priority group in the World Health Organisations Global strategy on Alcohol[1]. Concerns about the use of alcohol among young people have been rising over the last decade with young people the age group most likely to consume alcohol at levels of high risk. [2 3] Whilst recent Australian survey data has indicated that adolescents who are drinking at risky levels has declined this conflicts with increasing evidence of alcohol-related harm, particularly increases in alcohol-related hospital presentations by 16-17 year olds.[4] Alcohol-related violence is a growing problem among youth [5] and heavy drinking and intoxication is associated with violent offences.[6 7]

Given the strong links between alcohol use and violence, strategies to reduce violence need to address alcohol use. We have previously shown that violence-related injury accounts for almost a quarter of all injury admissions to hospital for adolescents aged 16 to 17 years.[8] Clinicians need information on the burden of alcohol-related injury admissions and the cause of those injuries in order to set in place services to address harmful drinking and reduce the risk of future injury or other health consequences. In addition, trends in alcohol-related injury admissions, and variation in trends between genders can inform policy about high risk groups. Variation in these patterns between countries contributes to evidence on potential service, policy, commercial or cultural influences on alcohol-related injury.

The aim of this study was to determine trends in alcohol-related injury admissions to hospital among adolescents in Western Australia and England and whether these

For peer review only - http://bmjopen.bmj.com/site/about/guidelines.xhtml

admissions are violence-related. Western Australia and England have similar health systems and have comparable rates of average daily alcohol consumption. [9] We also examined whether trends varied by gender and age-groups to determine groups most at risk. Our study focussed on adolescents aged 13-17 years as this is a priority age group for early intervention and prevention of harmful patterns of alcohol use.[10]

METHODS

Datasets

This study utilised hospital admissions data from England and Western Australia (WA). WA data included adolescents admitted for unplanned injury between 13 and 17 years of age to both private and public hospitals between 1990 and 2009. In England, we included the same age group for unplanned injury admissions to the National Health Service (NHS) in England between 1997 and 2012. In England each episode of care contains up to 20 diagnostic codes which contain external cause codes and in WA up to 21 diagnostic codes and 4 external cause codes. Both countries use International Classification of Diseases (ICD) coding systems with Western Australia moving from ICD-9 to ICD-10 on 1st July 1999, with England using only ICD-10 from 1997 onwards.

We defined injury admissions based on any diagnostic code for injury, poisoning or other consequences of external cause (ICD Codes: S or T). In WA ICD-10 and their equivalent codes in ICD-9 were used. Alcohol related injuries were identified if they contained ICD-10 diagnostic codes which have been used as identifiers for alcohol-related admissions in

For peer review only - http://bmjopen.bmj.com/site/about/guidelines.xhtml

previous studies [4 11]: F10, E24.4, G31.2, G62.1, G72.1 I42.1, K29.2, K70, K85.2, K86.0, R78.0, T50.6, T51, X45, X65, Y15, Y91, Z50.2, Z71.4, Z72.1.

Causes of injury were classified as intentional if codes were recorded for assault, (ICD-10: Y04, Y05, X85-Y03, Y08-Y09) maltreatment (ICD-10: T74, T73, Y06, Y07), self-harm, (ICD-10: X60-X69, X70-X84, Z91.5), or undetermined cause (ICD-10: Y20-Y34, Z04.5, Z04.8). The sub-group of violence-related injuries was defined by codes related to assault, maltreatment, or undetermined cause. Unintentional injuries were those injury admissions that contained codes of 'accident' as the external cause, including road traffic crashes (V01-V99, W00-X59).

Ethics

This study had ethics approval from the University of Western Australia Human Ethics Committee, the Department of Health Human Research Ethics Committee and the Western Australian Aboriginal Human Information and Ethics Committee. Use of anonymised data in England for the purpose in this study did not require research ethics approval but met the requirements of the data provider.[12]

Analyses

Annual incidence rates for maltreatment-related admissions were calculated by dividing the number of children who had these admissions by age-specific population estimates for each calendar year. In WA the denominator data was sourced from the Australian Bureau of Statistics population estimates (ABS, 2009). In England population denominators were based on mid-year population estimates obtained from the UK Office

For peer review only - http://bmjopen.bmj.com/site/about/guidelines.xhtml

of National Statistics website (UK National Statistics, 2009). Time trends in the annual incidence of admissions were examined using Poisson regression and percentage change per year reported including confidence intervals to indicate significance. Poisson has been recommended for quantifying time trends of discrete events such as injury admissions[13]. We compared absolute rates in 2009 as this was the most recent year for data common to both countries. SAS release 9.1 was used to conduct all analyses.[14]

RESULTS

Injury admissions for any type of injury have declined in both WA and England over the last 10 to 20 years. However despite this decrease WA hospital admissions for alcohol-related injuries for 13-17 year olds increased during 1990-2009 from 8.2 to 12 per 10,000, an increase of 2.3% (1.5, 3.1) per year. Conversely, in England, the rate of admissions for alcohol-related injuries decreased marginally from 9.1 to 6.5 per 10,000 people, over the period of available data from 1997 to 2012, although this was a non-significant decrease (1.1% per year, 95% CI -2.5, 0.4).

Age comparisons indicated that in WA there was an increase in alcohol-related injuries in the 16-17 year age group, an increase of 2.7% (1.9, 3.6) per year (from 14.3 to 22 per 10,000). In the 13-15 year age group there was a non-significant increase of 1.2% (-0.2, 2.5) per year (from 4 to 4.8 per 10,000). This is in contrast to England where rates for the 16-17 year age group had a non-significant increase of 1.4% (-0.3, 3.1) per year and the rate for 13-15 year olds decreased by 3.7% (-5.4, -1.9) per year.

For peer review only

For peer review only - http://bmjopen.bmj.com/site/about/guidelines.xhtml

Gender comparisons showed that in WA boys aged 16-17 years had the highest rate of alcohol-related injury admissions at 27 per 10,000 in 2009, while the largest change in trends was for girls across both the 13-15 and 16-17 age groups (Figure 1). The rates of alcohol-related injury admissions in the 13-15 year age group increased by 2.7% (0.9, 4.6) per year for girls and remained stable for boys (-0.4% (-1.9, 1.1) per year for boys. In the 16-17 year age group, girls also had a larger change in trends at 3.2% (2.1, 4.5) per year compared to boys at 2.5% (1.4, 3.5).

In England, there has been an overall increase of 4.1% (2.3, 6.0) per year in alcohol-related injury rates for girls aged 16-17 (from 9 to 12 per 10,000) whereas rates have declined for all other groups. For boys aged 13-15 there has been a significant decrease of 6.5% (-8.0, -5.0) per year. For girls aged 13-15 and boys aged 16-17 there have been non-significant decreases of 1.6% (-3.7, 0.6) and 0.8% (-2.5, 0.9) per year respectively. Alcohol-related injury rates in England have been decreasing for both genders since 2007, with the most recent rate in 2012 being much lower in England compared to Western Australia for boys aged 16-17 years.

In Western Australia, the predominant cause for alcohol-related injuries for 13-17 year old boys was unintentional, closely followed by intentional injuries. The opposite was true for 13-17 year old girls, where intentional injury rates were twice as high as unintentional injury rates. This difference was most notable among 16-17 year old girls where there were 11.5 per 10,000 intentional alcohol-related injuries in 2009 compared to 4.8 per 10,000 unintentional alcohol-related injuries (Figure 2). In England, the predominant cause of alcohol-related injury was intentional, with 16-17 year old girls in

For peer review only

particular showing the highest rates (10.6 per 10,000 in 2009). This was one of the few trends that mirrored the pattern shown in Western Australia.

Alcohol-related injuries that were recorded as intentional are shown according to whether records indicated violence (which were predominantly assaults) or self-harm (Figure 3). In Western Australia, alcohol-related injuries caused by violence increased for both genders. This is in contrast to England where violence related injury rates remained relatively stable. In WA violence-related injuries for boys aged 13-17 year old (4 per 10,000 in 2009) were the most prevalent intentional injuries, with the highest being for 16-17 year old boys at 8 per 10,000, increasing 4.7% (2.8, 6.7) per year. For girls 16-17 year olds have higher rates (2.7 per 10,000 in 2009) compared to 13-15 year old girls, however they remained stable from 1990-2009 but increased significantly for 13-15 year olds by 7.1% per year (Figure 3). Self-harm predominated among girls in both WA and England, showing increasing rates in both countries, but a decline in England since 2007.

Unintentional alcohol-related injury rates have also increased in Western Australia for both boys and girls between 1990 and 2009 apart from 13-15 year old boys which remained stable. In England, unintentional alcohol-related injury rates have generally fallen or remained stable across both genders.

Absolute rates of alcohol-related injuries are shown for both countries for 2009, the most recent year in which comparable data was available for Western Australia (Table 1). For 13-17 year olds overall, Western Australia had higher rates of alcohol-related injuries in all categories other than self-harm. In both countries, 16-17 year olds had the highest rate

For peer review only - http://bmjopen.bmj.com/site/about/guidelines.xhtml

of alcohol-related injuries, however the rate was twice as high among 16-17 year old boys in Western Australia (27.1/10,000) than in England (13.2/10,000). This pattern was consistent across both intentional and unintentional injuries. England had higher rates of alcohol-related self-harm, particularly among girls aged 13-15 (5.1/10,000) than Western Australia (2.5/10,000).

For peer review only

For peer review only - http://bmjopen.bmj.com/site/about/guidelines.xhtml

Table 1. Alcohol-related injury admission rates for children in Western Australia and England aged 13-17 years, 2009

Age group	Alcohol-related cause of injury	Western Australia			England		
		Boys<br>Absolute rate 2009<br>(per 10,000)	Girls<br>Absolute rate 2009<br>(per 10,000)	Total<br>Absolute rate 2009<br>(per 10,000)	Boys<br>Absolute rate 2009<br>(per 10,000)	Girls<br>Absolute rate 2009<br>(per 10,000)	Total<br>Absolute rate 2009<br>(per 10,000)
13-15 years	Injury	3.7 (2.3, 5.9)	6.0 (4.1, 8.8)	4.8 (3.6, 6.5)	4.6 (4.2, 5.1)	8.7 (8.1, 9.3)	6.6 (6.3, 7.0)
	Intentional	1.5 (0.7, 3.2)	3.7 (2.3, 6.0)	2.6 (1.7, 3.9)	1.8 (1.5, 2.1)	5.5 (5.0, 6.0)	3.6 (3.3, 3.9)
	Violence	0.9 (0.3, 2.3)	1.1 (0.5, 2.8)	1.0 (0.5, 1.9)	0.7 (0.5, 0.9)	0.5 (0.4, 0.7)	0.6 (0.5, 0.7)
	Self-harm	0.6 (0.2, 2.0)	2.5 (1.4, 4.6)	1.6 (0.9, 2.6)	1.1 (1.0, 1.4)	5.1 (4.7, 5.6)	3.1 (2.8, 3.3)
	Unintentional	2.2 (1.2, 4.0)	1.6 (0.8, 3.4)	1.9 (1.2, 3.0)	2.8 (2.5, 3.2)	3.6 (3.2, 4.0)	3.2 (2.9, 3.5)
16-17 years	Injury	27.1 (21.9, 33.5)	16.6 (12.6, 22.0)	22.0 (18.6, 26.1)	13.2 (12.4, 14.1)	14.1 (13.2, 15.0)	13.6 (13.0, 14.3)
	Intentional	12.9 (9.5, 17.6)	11.5 (8.3, 16.2)	12.2 (9.7, 15.4)	6.9 (6.3, 7.6)	10.6 (9.8, 11.5)	8.7 (8.2, 9.2)
	Violence	8.4 (5.7, 12.3)	2.7 (1.4, 5.4)	5.6 (4.0, 7.9)	2.9 (2.5, 3.3)	0.5 (0.4, 0.7)	1.7 (1.5, 2.0)
	Self-harm	4.8 (2.9, 8.0)	8.8 (6.0, 13.0)	6.8 (5.0, 9.2)	4.1 (3.7, 4.6)	10.2 (9.4, 11.0)	7.1 (6.6, 7.5)
	Unintentional	12.9 (9.5, 17.6)	4.8 (2.8, 8.0)	8.9 (6.8, 11.7)	6.5 (5.9, 7.1)	3.9 (3.4, 4.4)	5.2 (4.8, 5.6)

For peer review only - http://bmjopen.bmj.com/site/about/guidelines.xhtml

For peer review only

13-17 years	Injury	13.1 (10.8, 15.9)	10.3 (8.2, 12.9)	11.7 (10.1, 13.6)	8.2 (7.8, 8.7)	10.9 (10.4, 11.5)	9.5 (9.2, 9.9)
	Intentional	6.1 (4.6, 8.1)	6.9 (5.2, 9.0)	6.5 (5.3, 7.9)	3.9 (3.6, 4.2)	7.6 (7.2, 8.0)	5.7 (5.4, 6.0)
	Violence	3.9 (2.7, 5.6)	1.8 (1.0, 3.1)	2.9 (2.1, 3.9)	1.6 (1.4, 1.8)	0.5 (0.4, 0.6)	1.1 (1.0, 1.2)
	Self-harm	2.3 (1.5, 3.7)	5.1 (3.7, 7.0)	3.7 (2.8, 4.8)	2.4 (2.2, 2.6)	7.2 (6.8, 7.7)	4.7 (4.5, 5.0)
	Unintentional	6.5 (4.9, 8.5)	2.9 (1.9, 4.4)	4.7 (3.7, 6.0)	4.3 (4.0, 4.7)	3.7 (3.4, 4.0)	4.0 (3.8, 4.2)

For peer review only

For peer review only - http://bmjopen.bmj.com/site/about/guidelines.xhtml

Further analysis of the alcohol-related injury admission was conducted for Western Australia. Overall, the average length of stay associated with alcohol-related admissions was 2.6 days. We found that of self-harm injuries in boys, 24% were alcohol-related, compared to 13% of self-harm injuries in girls. For both genders, 15-17% of assault injuries were alcohol-related, compared to only 3% of accidental injuries. For 16-17 year olds alcohol was associated with 18% of assault-related injuries compared to 12% in 13-15 year olds. In 2009 this was even higher at 27% of assault-related injuries being associated with alcohol in the 16-17 year old age group. The predominant type of alcohol-related assault was assault by bodily force at (53%), followed by assault by unspecified means (18%), assault by blunt object (14%) and assault by sharp object (10%). In 3% of alcohol-related assault admissions the assault was rape.

DISCUSSION

Despite the decline in hospital admissions in Western Australia from 1990 to 2009 for injury for adolescents aged 13-17 there has been an increase in alcohol-related injuries by 2.3% per year. This increase has been largest in the 16-17 year old age group with boys aged 16-17 years having the highest rates. However girls in both the 13-15 and 16-17 age groups had the greatest increase in trends of 2.7% and 3.2% per year respectively. This substantiates and builds on a previous Australian study showing increases in alcohol-related injury admissions in 16-17 year olds till 2005 and a large rise in hospital presentations for girls.[4]

For peer review only - http://bmjopen.bmj.com/site/about/guidelines.xhtml

In contrast to the increasing rates of alcohol-related injuries in Western Australia, England's rates remained relatively stable over the study period. Most age and gender groups have shown decreasing or stable rates of alcohol-related injuries since 2007, with the exception of 16-17 year old girls. This suggests that alcohol-related harm in adolescents is a problem that can be reduced. Although coding practices can affect the magnitude of the trend, a decrease in overall alcohol-related harms was found after adjusting for coding changes [15], and surveys of 11-15 year olds from 2001-2011 also show a decrease in frequent drinking.[16] Recent data from Public Health England has also found a 13.9% fall in hospital admissions caused by alcohol in England for under 18's which corroborates our findings. [17]

In England, self-harm was the leading cause of alcohol-related injury particularly among girls, an issue which warrants further attention. Self-harm was also the most common form of intentional alcohol-related injury for girls in Western Australia. Research has found many adolescents admitted for injuries with self-harm also had drug or alcohol misuse coded and increased likelihood of having experienced violence, suggesting a need for strategies that address multiple forms of adversity. [18]

A large minority of injuries in Western Australia were unintentional injuries however there was a rise in violence-related admissions. Overall alcohol was associated with 16% of assault-related admissions and this was higher in 2009 for adolescents aged 16-17 years, with 20% of assaults associated with alcohol for girls and 30% for boys. This highlights the growing issue in Australia with the rise in violent injuries related to alcohol and the associated harm that adolescents are experiencing. It is important that this issue

For peer review only - http://bmjopen.bmj.com/site/about/guidelines.xhtml

be monitored, firstly so we know the extent of harm and the burden on the health system of alcohol related injuries and secondly that as strategies are implemented to reduce this burden we can look at the impact on the rate of admissions.

Unfortunately our data only captures when the individual admitted for the injury has an alcohol-related diagnosis not when the perpetrator had been affected by alcohol. Therefore this would certainly underestimate the rates of injury associated with alcohol use which is also important in capturing the burden. Research suggests alcohol use by the perpetrator was a factor in over half of physical and sexual assaults,[19] and has established associations with child maltreatment [20 21] and road traffic crashes.[22] Future research could use data from police and corrective services to investigate alcohol-related harm. Also our data only relates to injuries requiring hospitalisation not those seen in the Emergency Departments (ED) therefore our injury admissions would relate to more serious injuries and violence. Another limitation is that changes in coding over time may influence apparent trends. Our study shows that at the least in WA there is an increase in recognition (whether due to more rigorous reporting or as a rise in actual incidents) of the influence of alcohol in injury admissions.

Using hospital admissions data allows monitoring of trends over time and country comparisons can generate hypotheses about policies or service factors that may account for differences. This is the first study examining and comparing trends in alcohol-related harm in adolescents in Western Australia and England. Identification of age and gender groups at greatest risk, and the types of harm they experience is necessary to inform public health initiatives targeting alcohol-related harm in adolescents. In England It is

For peer review only - http://bmjopen.bmj.com/site/about/guidelines.xhtml

unclear which specific policies or practices have influenced the decline, but a strategy for reducing alcohol-related harm has been in place since 2004. In 2008, an annual tax escalator was implemented to boost duties on all alcohol, and an agreement was signed to prevent alcohol branding on children's replica sports shirts.[23] Challenge 21 and subsequently Challenge 25, requiring identification from people who look less than 21 or 25 years old when purchasing alcohol, have also been introduced. Researchers have also stated that the economic downturn in England and the rise in alcohol taxation seemed to have stemmed the rise in alcohol-related deaths and in our research potentially alcohol-related injuries[24]. English researchers have also noted falls in other adversity-related admissions from 2005-11 and discussed the implementation of programs (eg. Sure Start, Alcohol Misuse Enforcement Campaign) which may have influenced rates[25]. Although progress is being made in implementing pricing strategies, many public health professionals and researchers advocate for greater changes in pricing policy, regulation and availability of treatment programs in order to reduce alcohol-related harm and deaths.[23]

Some strategies and programs are in place in Australia to address youth drinking and alcohol-related harm.[2 3] These appear to be having some positive effects, with adolescents being the only group that has shown a decrease in the proportion of people who drink at risky levels.[3] Nonetheless, our research shows that rates of alcohol-related injury admissions increased in Western Australia, consistent with findings for Victoria, Australia.[4] Possible explanations for this discordance include the hypothesis that sub-groups excluded from surveys (such as homeless or truant youth, those who left school

For peer review only - http://bmjopen.bmj.com/site/about/guidelines.xhtml

prior to Year 12 for school surveys, or those that chose not to participate) have had increased drinking or that sub-groups have increased excessive and risky drinking.[26]

Australia and England follow a number of the World Health Organisation recommendations for reducing alcohol-related harm including maximum blood alcohol levels while driving, and restrictions on age, sales to intoxicated people, and promotion of alcoholic beverages at activities targeting young people.[9] However more clearly needs to be done to reduce the rising rates of alcohol-related harm in Western Australia and in England despite the declining rates Professor Kevin Fenton, Director of Health and Wellbeing at Public Health England has stated that 'while this is promising… current levels of harm caused by alcohol remain unacceptably high, especially within the most deprived communities, who suffer the most from poor health in general.'[27]

Evidence suggests the optimal package of interventions also includes measures to reduce the affordability and availability of alcohol.[28-30] There is evidence that pricing policy can significantly reduce alcohol consumption [31 32] and alcohol-related hospital admissions [30] and deaths [33]. Minimum unit pricing or volumetric pricing are considered effective and cost-effective solutions.[34] In 2012, Scotland followed Canada in legislating for minimum alcohol unit pricing.[35] Recent research has found the majority of young risky drinkers support evidence-based policies including price increases if revenue is used to support treatment and prevention of alcohol problems.[36]

For peer review only

For peer review only - http://bmjopen.bmj.com/site/about/guidelines.xhtml

CONTRIBUTORSHIP STATEMENT

All authors have contributed to the conceptualisation of this paper and/or to the writing of this paper and/or contributed to the analysis of data and its interpretation.

COMPETING INTERESTS

The authors have no competing interests.

FUNDING AND ACKNOWLEDGEMENTS

The Policy Research Unit in the Health of Children, Young People and Families (CPRU), which is funded by the England Department of Health Policy Research Programme. This is an independent report commissioned and funded by the Department of Health. We thank members of CPRU: Terence Stephenson, Catherine Law, Amanda Edwards, Steve Morris, Helen Roberts, Cathy Street, Russell Viner and Miranda Wolpert. RG and AGI were also partly supported by awards establishing the Farr Institute of Health Informatics Research (MR/K006584/1). Melissa O'Donnell was supported by a National Health and Medical Research Council Early Career Fellowship [1012439]. This research was also supported by an Australian Research Council Linkage Project Grant [LP100200507] and an Australian Research Council Discovery Grant [DP110100967]. We also acknowledge the partnership of the Western Australian Government Departments of Health, Child Protection, Education, Disability Services, Corrective Services and the Attorney General, which provided data for this project. The views expressed are not necessarily those of the Government Departments that have commissioned or supported this project.

DATA SHARING STATEMENT

The data utilised in this paper is owned by our respective government departments and therefore would require permissions by these departments for others to access.

For peer review only

REFERENCES

1. Degenhardt L, Stockings E, Patton G, et al. The increasing global health priority of substance use in young people. The Lancet 2016;**Published online 18th February 2016**.

2. Moodie R, Daube M, Carnell K, et al. Australia: The healthiest country by 2020 National Preventative Health Strategy - the roadmap for action. Canberra Australian Government Preventative Health Taskforce, 2009.

3. Moodie R, Daube M, Zimmet P, et al. Taking Preventative Action: A Response to Australia: The Healthiest Country by 2020 - The Report of the National Preventative Health Task Force. Canberra: Australian Government Preventative Health Taskforce, 2010.

4. Livingston M. Recent trends in risky alcohol consumption and related harm among young people in Victoria, Australia. Australian and New Zealand Journal of Public Health 2008;**32**(3):266-71.

5. Morgan A, McAtamney A. Key issues in alcohol-related violence. Research in Practice: Summary no. 4. . Canberra: Australian Institute of Criminology, 2009.

6. Plant M, Plant M, Thornton C. People and places: Some factors in the alcohol-violence link. Journal of Substance Use 2002;**7**(4):207-13.

7. Krug EG, Dahlberg LL, Mercy JA, et al. World report on violence and health. Geneva: World Health Organization, 2002.

8. Gonzalez-Izquierdo A, Ward A, O'Donnell M, et al. Cross-country comparison of victimisation-related injury admission in children and adolescents in England and Western Australia. BMC Health Services Research 2013;**13**(1):260.

For peer review only - http://bmjopen.bmj.com/site/about/guidelines.xhtml

9. WHO. Global status report on alcohol and health 2014 Geneva: World Health Organization, 2014.

10. Toumbourou JW, Stockwell T, Neighbors C, et al. Adolescent Health 4: Interventions to reduce harm associated with adolescent substance use. Lancet 2007;**369**(9570):1391-401.

11. Egerton-Warburton D, Gosbell A, Wadsworth A, et al. Survey of alcohol-related presentations to Australasian emergency departments. Medical Journal of Australia 2014;**201**(10):584-87.

12. NHS. The information centre for health and social care. HES online. The approvals process. 2012. 6-8-2012.

13. Kuhn L, Davidson LL, Durkin MS. Use of poisson regression and time series analysis for detecting changes over time in rates of child injury following a prevention program. American Journal of Epidemiology 1994;**140**(10):943-55.

14. SAS Version 9.1 [program]. Cary NC: SAS Institute Inc., 2004.

15. Lifestyle Statistics HaSCIC. Statistics on Alcohol: England, 2013, 2013.

16. Fuller E, Gill V, Hawkins V, et al. Smoking, drinking and drug use among young people in England in 2011, NatCen Social Research. London, 2012.

17. Public Health England. Local Alcohol Profiles for England, June 2015. Statistical Bulletin. London: Public Health England, 2015.

18. Herbert A, Gilbert R, González-Izquierdo A, et al. Violence, self-harm and drug or alcohol misuse in adolescents admitted to hospitals in England for injury: a retrospective cohort study. BMJ Open 2015;**5**(2):e006079.

For peer review only

For peer review only - http://bmjopen.bmj.com/site/about/guidelines.xhtml

19. Connor J, You R, Casswell S. Alcohol-related harm to others: a survey of physical and sexual assault in New Zealand. New Zealand Medical Journal 2009;**122**(1303).

20. Gilbert R, Widom CS, Browne K, et al. Burden and consequences of child maltreatment in high-income countries. Lancet 2009;**373**(9657):68-81.

21. Laslett A-M, Ferris J, Dietze P, et al. Social demography of alcohol-related harm to children in Australia. Addiction 2012;**107**(6):1082-89.

22. Bramwell J, Hill D, Thompson PE. Reported road crashes in Western Australia 2012. Perth: Road Safety Council of Western Australia, 2014.

23. Gilmore I, Anderson W, Bauld L, et al. Health first: An evidence-based alcohol strategy for the UK Stirling: University of Stirling, 2013.

24. Sheron N, Gilmore I. Effect of policy, econmics, and the changing alcohol marketplace on alcohol related deaths in England and Wales. British Medical Journal 2016;**353**:i1860

25. Herbert A, Gonzalez-Izquierdo A, McGhee J, et al. Time-trends in rates of hospital admissions of adolescents for violent, self-inflicted or drug/alcohol-relted injury in England and Scotland, 2005-11: population-based analysis. Journal of Public Health 2016;**doi:10.1093/pubmed/fdw020**.

26. Livingston M, Matthews S, Barratt MJ, et al. Diverging trends in alcohol consumption and alcohol-related harm in Victoria. Australian and New Zealand Journal of Public Health 2010;**34**(4):368-73.

27. Public Health England. Alcohol-related hospital admissions decrease among under 18s. London: Public Health England, 2015.

For peer review only

For peer review only - http://bmjopen.bmj.com/site/about/guidelines.xhtml

28. Anderson P, Chisholm D, Fuhr DC. Alcohol and global health 2: Effectiveness and cost-effectiveness of policies and programmes to reduce the harm caused by alcohol. Lancet 2009;**373**(9682):2234-46.

29. Casswell S, Thamarangsi T. Alcohol and global health 3: Reducing harm from alcohol: call to action. Lancet 2009;**373**(9682):2247-57.

30. Stockwell T, Zhao J, Martin G, et al. Minimum alcohol prices and outlet densities in British Columbia, Canada: Estimated impacts on alcohol-attributable hospital admissions. American Journal of Public Health 2013;**103**(11):2014-20.

31. Wagenaar AC, Salois MJ, Komro KA. Effects of beverage alcohol price and tax levels on drinking: a meta-analysis of 1003 estimates from 112 studies. Addiction 2009;**104**(2):179-90.

32. Stockwell T, Zhao J, Giesbrecht N, et al. The raising of minimum alcohol prices in Saskatchewan, Canada: Impacts on consumption and implications for public health. American Journal of Public Health 2012;**102**(12):e103-e10.

33. Chikritzhs T, Stockwell T, Pascal R. The impact of the Northern Territory's Living With Alcohol program, 1992–2002: revisiting the evaluation. Addiction 2005;**100**(11):1625-36.

34. Sharma A, Vandenberg B, Hollingsworth B. Minimum pricing of alcohol versus volumetric taxation: Which policy will reduce heavy consumption without adversely affecting light and moderate consumers? PLoS ONE 2014;**9**(1):1-13.

35. Leicester A. Alcohol pricing and taxation policies, IFS Briefing Note BN124: Institute for Fiscal Studies, 2011.

For peer review only - http://bmjopen.bmj.com/site/about/guidelines.xhtml

36. Lam T, Lenton S, Burns L, et al. Alcohol policy impact on young risky drinkers and their support for proposed measures. Australian and New Zealand Journal of Public Health 2015;**39**(2):12-134.

For peer review only

For peer review only - http://bmjopen.bmj.com/site/about/guidelines.xhtml

Figure 1. Rate of hospital admissions for alcohol-related injury in Western Australia and England (per 10,000 persons) for adolescents aged 13-15 and 16-17years

Western Australia **England**

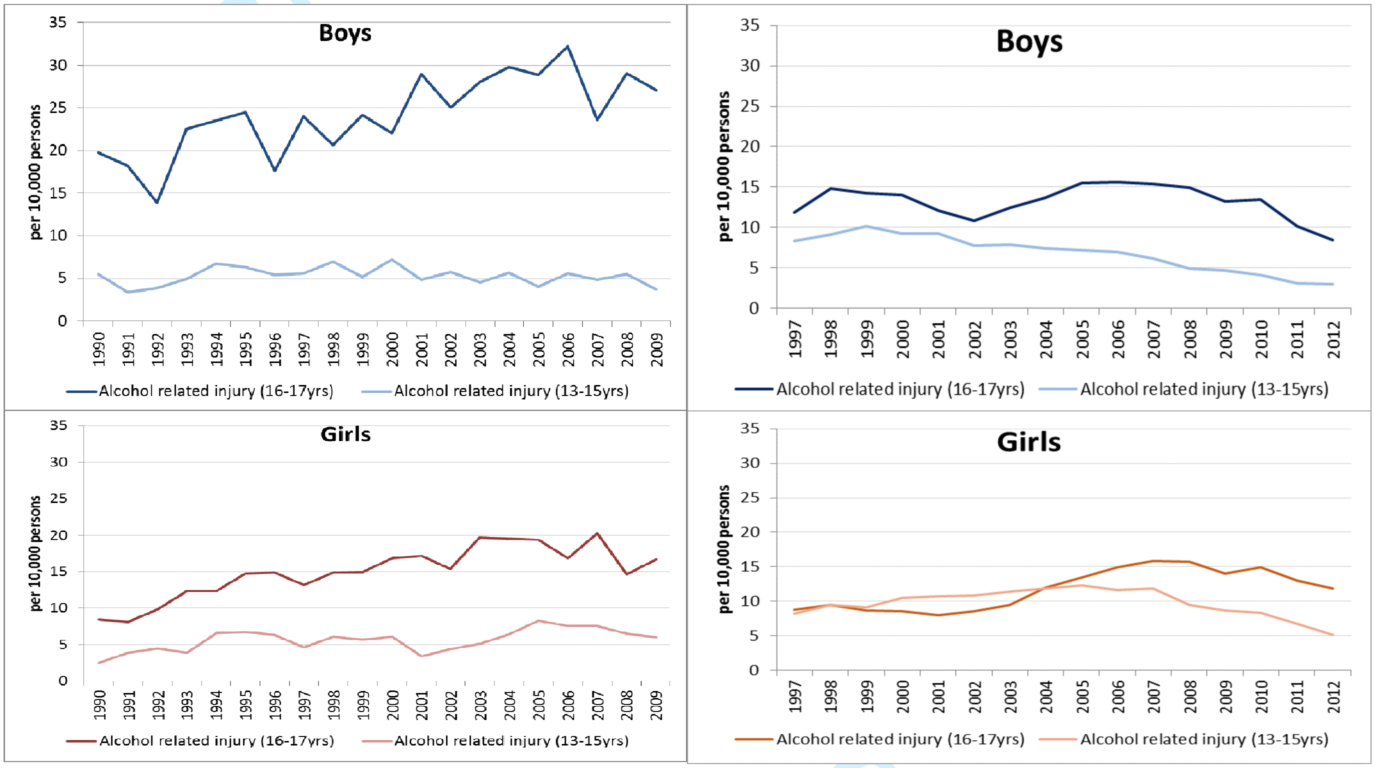

For peer review only - http://bmjopen.bmj.com/site/about/guidelines.xhtml

Figure 2. Rate of intentional and unintentional alcohol-related injury hospital admissions in Western Australia and England (per 10,000 persons) for adolescents aged 13-15 and 16-17years

Western Australia **England**

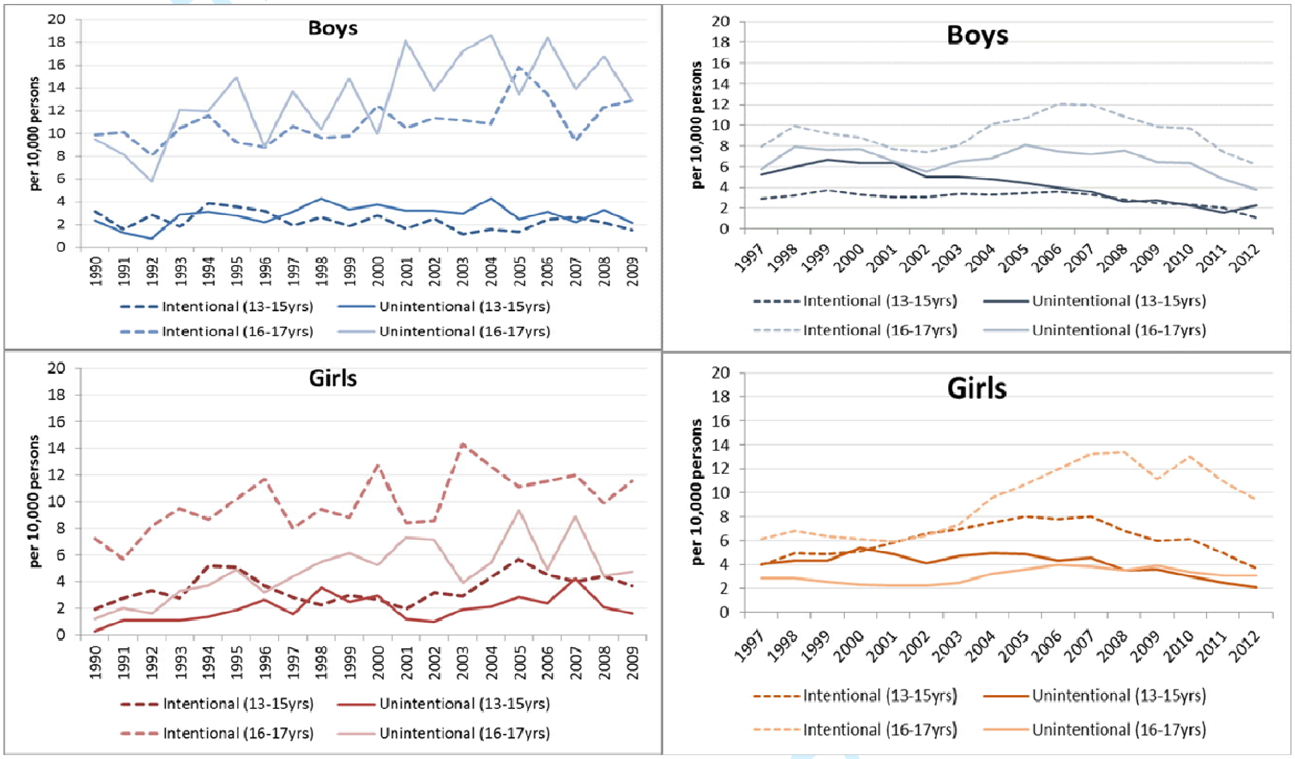

For peer review only - http://bmjopen.bmj.com/site/about/guidelines.xhtml

Figure 3. Rate of intentional alcohol-related injury hospital admissions in Western Australia and England (per 10,000 persons) for adolescents aged 13-17 years, by violence-related and self-harm admissions

Western Australia **England**

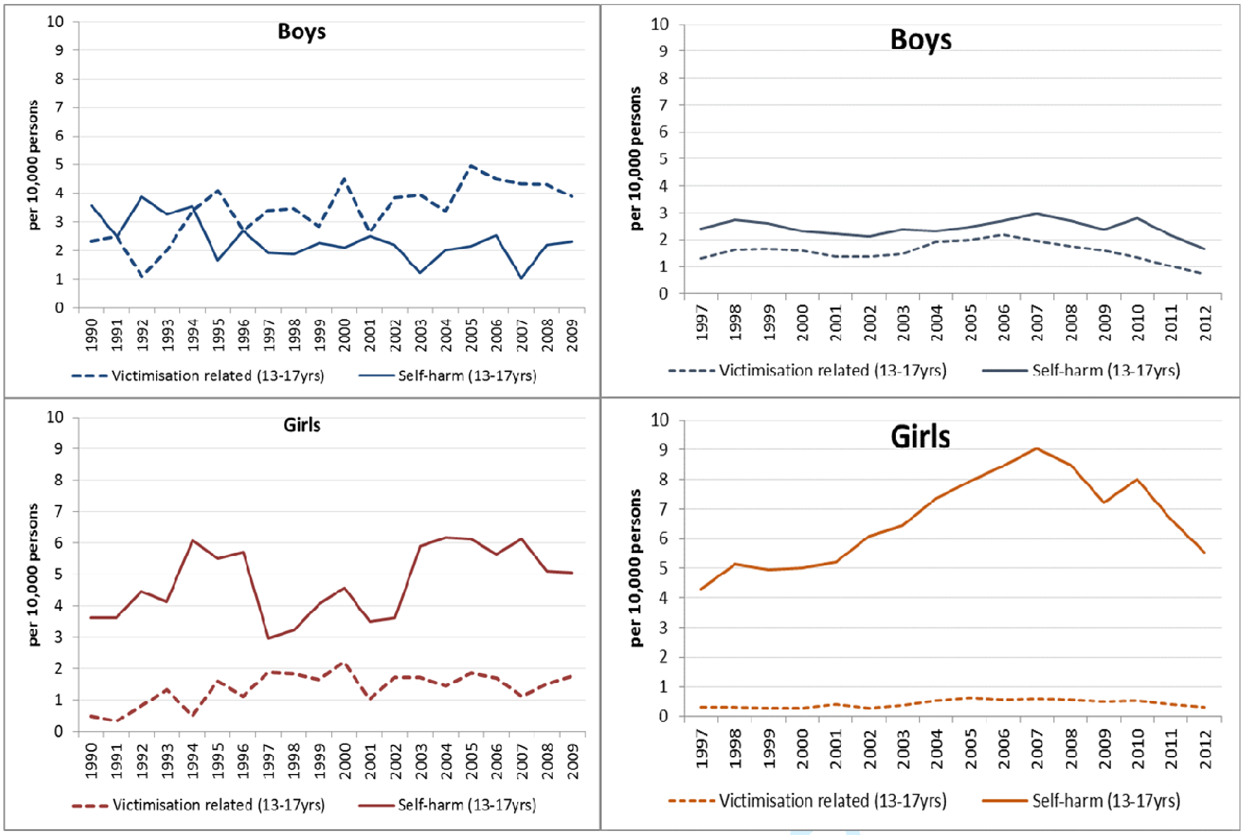

only

For peer review only - http://bmjopen.bmj.com/site/about/guidelines.xhtml

STROBE Statement—checklist of items that should be included in reports of observational studies

	Item No	Recommendation
**Title and abstract**	1 – a and b completed	(*a*) Indicate the study's design with a commonly used term in the title or the abstract
		(*b*) Provide in the abstract an informative and balanced summary of what was done and what was found
**Introduction**		
Background/rationale	2 completed	Explain the scientific background and rationale for the investigation being reported
Objectives	3 completed	State specific objectives, including any prespecified hypotheses
**Methods**		
Study design	4 completed	Present key elements of study design early in the paper
Setting	5 completed	Describe the setting, locations, and relevant dates, including periods of recruitment, exposure, follow-up, and data collection
Participants	6 -population based cohort so not necessary to discuss selection criteria	(*a*) *Cohort study*—Give the eligibility criteria, and the sources and methods of selection of participants. Describe methods of follow-up<br>*Case-control study*—Give the eligibility criteria, and the sources and methods of case ascertainment and control selection. Give the rationale for the choice of cases and controls<br>*Cross-sectional study*—Give the eligibility criteria, and the sources and methods of selection of participants
		(*b*) *Cohort study*—For matched studies, give matching criteria and number of exposed and unexposed<br>*Case-control study*—For matched studies, give matching criteria and the number of controls per case
Variables	7- completed	Clearly define all outcomes, exposures, predictors, potential confounders, and effect modifiers. Give diagnostic criteria, if applicable
Data sources/ measurement	8*-completed	For each variable of interest, give sources of data and details of methods of assessment (measurement). Describe comparability of assessment methods if there is more than one group
Bias	9- completed	Describe any efforts to address potential sources of bias
Study size	10- Not Applicable	Explain how the study size was arrived at
Quantitative variables	11- completed	Explain how quantitative variables were handled in the analyses. If applicable, describe which groupings were chosen and why
Statistical methods	12 – a) b) c) Completed d) Not applicable	(*a*) Describe all statistical methods, including those used to control for confounding
		(*b*) Describe any methods used to examine subgroups and interactions
		(*c*) Explain how missing data were addressed
		(*d*) *Cohort study*—If applicable, explain how loss to follow-up was addressed<br>*Case-control study*—If applicable, explain how matching of cases and controls was addressed<br>*Cross-sectional study*—If applicable, describe analytical methods taking account of sampling strategy
		(*e*) Describe any sensitivity analyses

For peer review only

For peer review only - http://bmjopen.bmj.com/site/about/guidelines.xhtml

**Results**		
Participants	13*-completed	(a) Report numbers of individuals at each stage of study—eg numbers potentially eligible, examined for eligibility, confirmed eligible, included in the study, completing follow-up, and analysed
		(b) Give reasons for non-participation at each stage
		(c) Consider use of a flow diagram
Descriptive data	14*-completed	(a) Give characteristics of study participants (eg demographic, clinical, social) and information on exposures and potential confounders
		(b) Indicate number of participants with missing data for each variable of interest
		(c) *Cohort study*—Summarise follow-up time (eg, average and total amount)
Outcome data	15*-completed	*Cohort study*—Report numbers of outcome events or summary measures over time
		Case-control study—Report numbers in each exposure category, or summary measures of exposure
		Cross-sectional study—Report numbers of outcome events or summary measures
Main results	16-completed	(*a*) Give unadjusted estimates and, if applicable, confounder-adjusted estimates and their precision (eg, 95% confidence interval). Make clear which confounders were adjusted for and why they were included
		(*b*) Report category boundaries when continuous variables were categorized
		(*c*) If relevant, consider translating estimates of relative risk into absolute risk for a meaningful time period
Other analyses	17-completed	Report other analyses done—eg analyses of subgroups and interactions, and sensitivity analyses
**Discussion**		
Key results	18-completed	Summarise key results with reference to study objectives
Limitations	19-completed	Discuss limitations of the study, taking into account sources of potential bias or imprecision. Discuss both direction and magnitude of any potential bias
Interpretation	20-completed	Give a cautious overall interpretation of results considering objectives, limitations, multiplicity of analyses, results from similar studies, and other relevant evidence
Generalisability	21-completed	Discuss the generalisability (external validity) of the study results
**Other information**		
Funding	22 -completed	Give the source of funding and the role of the funders for the present study and, if applicable, for the original study on which the present article is based

*Give information separately for cases and controls in case-control studies and, if applicable, for exposed and unexposed groups in cohort and cross-sectional studies.

Note: An Explanation and Elaboration article discusses each checklist item and gives methodological background and published examples of transparent reporting. The STROBE checklist is best used in conjunction with this article (freely available on the Web sites of PLoS Medicine at http://www.plosmedicine.org/, Annals of Internal Medicine at http://www.annals.org/, and Epidemiology at http://www.epidem.com/). Information on the STROBE Initiative is available at www.strobe-statement.org.

For peer review only

For peer review only - http://bmjopen.bmj.com/site/about/guidelines.xhtml

BMJ Open

Trends in alcohol-related injury admissions in adolescents in Western Australia and England: Population-based cohort study

Journal:	*BMJ Open*
Manuscript ID	bmjopen-2016-014913.R1
Article Type:	Research
Date Submitted by the Author:	22-Jan-2017
Complete List of Authors:	O'Donnell, Melissa; University of Western Australia, Telethon Kids Institute<br>Sims, Scott; Telethon Kids Institute, University of Wester Australia,<br>Maclean, Miriam; Telethon Kids Institute, University of Western Australia,<br>Gonzalez-Izquierdo, Arturo; University College London<br>Gilbert, Ruth; University College London, Institute of Child Health<br>Stanley, Fiona; University of Western Australia, Telethon Kids Institute
**Primary Subject Heading**:	Public health
Secondary Subject Heading:	Epidemiology, Public health
Keywords:	alcohol, adolescents, EPIDEMIOLOGY, Non-accidental injury < PAEDIATRICS

SCHOLARONE™
Manuscripts

For peer review only

Running Head: TRENDS IN ALCOHOL-RELATED INJURY ADMISSIONS

Title: Trends in alcohol-related injury admissions in adolescents in Western Australia and England: Population-based cohort study

Melissa O'Donnell, BPsych (Hons), MPsych, GradDip Ed, PhD, NHMRC Postdoctoral Research Fellow, Telethon Kids Institute, University of Western Australia, Perth, Australia. Email: melissao@ichr.uwa.edu.au

Scott Sims, BSc, MBiostat, Data Analyst, Telethon Kids Institute, University of Western Australia, Perth, Australia. Email: Scott.Sims@telethonkids.org.au

Miriam J Maclean, BPsych (Hons), MSc, Telethon Kids Institute, University of Western Australia, Perth, Australia. Email: Miriam.Maclean@telethonkids.org.au

Arturo Gonzalez-Izquierdo, PhD, Data Scientist, Farr Institute of Health Informatics Research, University College London, London. Email: arturo.gonzalez-izquierdo@ucl.ac.uk

Ruth Gilbert, MD, MSc, Professor of Clinical Epidemiology, Population Policy and Practice Programme, University College London, Institute of Child Health, London. Email: r.gilbert@ucl.ac.uk

For peer review only

For peer review only - http://bmjopen.bmj.com/site/about/guidelines.xhtml

Fiona J Stanley, MD, MSc, FFPHM, FAFPHM, Research Professor, Telethon Kids Institute, University of Western Australia, Perth, Australia. Email: Fiona.Stanley@telethonkids.org.au

Corresponding Author and Address: Melissa O'Donnell, Telethon Kids Institute, University of Western Australia, 100 Roberts Road, Subiaco, Western Australia. Telephone: (08) 9489 7778. Email: Melissa.O'Donnell@telethonkids.org.au

For peer review only

Keywords: Injury rates, trends, alcohol, adolescents, youth

Number of Tables=1

Number of Figures=3

Word Count=3110

For peer review only - http://bmjopen.bmj.com/site/about/guidelines.xhtml

Abstract

Background: Alcohol-related harm in young people is now a global health priority. We examined trends in hospital admissions for alcohol-related injuries for adolescents in Western Australia (WA) and in England, identified groups most at risk and determined causes of injuries.

Methods: Annual incidence rates for alcohol-related injury rates were calculated using population-level hospital admissions data for WA and England. We compared trends in different types of alcohol-related injury by age and gender.

Results: Despite a decrease in the overall rate of injury admissions for 13-17 year olds in WA, alcohol-related injuries have increased significantly from 1990-2009 (from 8 to 12 per 10,000). Conversely, alcohol-related injury rates have declined in England since 2007. In England self-harm is the most frequently recorded cause of alcohol-related injury. In WA, unintentional injury is most common, however violence-related harm is increasing for boys and girls.

Conclusions: Alcohol-related harm of sufficient severity to require hospital admission is increasing among adolescents in Western Australia. Declining trends in England suggests this trend is not inevitable or irreversible. More needs to be done to address alcohol-related harm, and ongoing monitoring is required to assess the effectiveness of strategies.

Strengths and limitations of this study

- This study investigates trends in adolescent hospital admissions for injuries and the documented causes of these injuries.
- While trends in alcohol-related admissions has been reported in previous research this adds to the knowledge of trends in different age groups and causes of injury.

For peer review only - http://bmjopen.bmj.com/site/about/guidelines.xhtml

- The limitations of this research is that only individuals admitted for injuries who have an alcohol-related diagnosis will be captured not when the perpetrator had been affected by alcohol therefore underestimating rates of injury associated with alcohol use.
- There also could be changes in coding practices which may contribute to changes in trends.

For peer review only

For peer review only - http://bmjopen.bmj.com/site/about/guidelines.xhtml

INTRODUCTION

Substance use in young people is considered a global health priority with young people highlighted as a priority group in the World Health Organisations Global strategy on Alcohol[1]. Concerns about the use of alcohol among young people have been rising over the last decade with young people the age group most likely to consume alcohol at levels of high risk. [2 3] Whilst recent Australian survey data has indicated that adolescents who are drinking at risky levels has declined this conflicts with increasing evidence of alcohol-related harm, particularly increases in alcohol-related hospital presentations by 16-17 year olds.[4] Alcohol-related violence is a growing problem among youth [5] and heavy drinking and intoxication is associated with violent offences.[6 7]

Given alcohol use at harmful levels is a significant risk factor for violence, strategies to reduce violence need to address alcohol misuse[8 9]. We have previously shown that violence-related injury accounts for almost a quarter of all injury admissions to hospital for adolescents aged 16 to 17 years.[10] Clinicians need information on the burden of alcohol-related injury admissions and the cause of those injuries in order to set in place services to address harmful drinking and reduce the risk of future injury or other health consequences. In addition, trends in alcohol-related injury admissions, and variation in trends between genders can inform policy about high risk groups. Variation in these patterns between countries contributes to evidence on potential service, policy, commercial or cultural influences on alcohol-related injury.

The aim of this study was to determine trends in alcohol-related injury admissions to hospital among adolescents in Western Australia and England and whether these

For peer review only

admissions are violence-related. Western Australia and England have similar health systems and have comparable rates of average daily alcohol consumption. [11] We also examined whether trends varied by gender and age-groups to determine groups most at risk. Our study focussed on adolescents aged 13-17 years as this is a priority age group for early intervention and prevention of harmful patterns of alcohol use.[12]

METHODS

Datasets

This study utilised hospital admissions data from England and Western Australia (WA). WA data included adolescents with injury admissions between 13 and 17 years of age to both private and public hospitals between 1990 and 2009. In England, we included the same age group for injury admissions to the National Health Service (NHS) in England between 1997 and 2012. Admissions were excluded if they were planned/scheduled admissions. Therefore admissions were included if patients were admitted through the hospitals admissions process for a period of care (for any length of time) due to a sudden health issue. In England each episode of care contains up to 20 diagnostic codes which contain external cause codes and in WA up to 21 diagnostic codes and 4 external cause codes. Both countries use International Classification of Diseases (ICD) coding systems with Western Australia moving from ICD-9 to ICD-10 on 1st July 1999, with England using only ICD-10 from 1997 onwards.

We defined injury admissions based on any diagnostic code for injury, poisoning or other consequences of external cause (ICD Codes: S or T). In WA ICD-10 and their equivalent

For peer review only - http://bmjopen.bmj.com/site/about/guidelines.xhtml

For peer review only

codes in ICD-9 were used. Alcohol-related injuries were identified if they contained ICD-10 diagnostic codes which have been used as identifiers for alcohol-related admissions in previous studies [4 13]: F10, E24.4, G31.2, G62.1, G72.1 I42.1, K29.2, K70, K85.2, K86.0, R78.0, T50.6, T51, X45, X65, Y15, Y91, Z50.2, Z71.4, Z72.1.

Causes of injury were classified as intentional if codes were recorded for assault, (ICD-10: Y04, Y05, X85-Y03, Y08-Y09) maltreatment (ICD-10: T74, T73, Y06, Y07), self-harm, (ICD-10: X60-X69, X70-X84, Z91.5), or undetermined cause (ICD-10: Y20-Y34, Z04.5, Z04.8). The sub-group of violence-related injuries was defined by codes related to assault, maltreatment, or undetermined cause which have been used in previous work[14]. Undetermined cause was included as they are an indication of potential assault and self-harm, however they only make up a small percent of our intentional injuries (<2%) and alcohol-related injuries (<1%). If individuals had codes in multiple categories (e.g. assault and self-harm) they were included in each category. Unintentional injuries were those injury admissions that contained codes of 'accident' as the external cause, including road traffic crashes (V01-V99, W00-X59).

Ethics

This study had ethics approval from the University of Western Australia Human Ethics Committee, the Department of Health Human Research Ethics Committee and the Western Australian Aboriginal Human Information and Ethics Committee. Use of anonymised data in England for the purpose in this study did not require research ethics approval but met the requirements of the data provider.[15]

For peer review only - http://bmjopen.bmj.com/site/about/guidelines.xhtml

Analyses

Annual incidence rates for maltreatment-related admissions were calculated by dividing the number of children who had these admissions by age-specific population estimates for each calendar year. In WA the denominator data was sourced from the Australian Bureau of Statistics population estimates (ABS, 2009). In England population denominators were based on mid-year population estimates obtained from the UK Office of National Statistics website (UK National Statistics, 2009). Time trends in the annual incidence of admissions were examined using Poisson regression and percentage change per year reported including confidence intervals to indicate significance. Poisson regression has been recommended for quantifying time trends of discrete events such as injury admissions[16]. All trends are presented as effect sizes with 95% confidence intervals (CI) and associated p-values and were considered statistically significant at the $p<0.05$ level if the CI did not include zero. A statistically non-significant trend ($p>0.05$) represents an inconclusive result. Due to some injury categories being relatively rare, there may be an effect which is undetected due to low sample sizes, thus it is still important to consider effect sizes and confidence intervals of trends despite non-significant p-values. We compared absolute rates in 2009 as this was the most recent year for data common to both countries. SAS release 9.1 was used to conduct all analyses.[17]

RESULTS

Injury admissions for any type of injury have declined in both WA and England over the last 10 to 20 years. However despite this decrease WA hospital admissions for alcohol-related injuries for 13-17 year olds increased during 1990-2009 from 8.2 to 12 per 10,000, an increase of 2.3% (95%CI:1.5, 3.1, $p<0.001$) per year. Conversely, in England,

For peer review only - http://bmjopen.bmj.com/site/about/guidelines.xhtml

the rate of admissions for alcohol-related injuries decreased marginally from 9.1 to 6.5 per 10,000 people, over the period of available data from 1997 to 2012, although this was a non-significant decrease (1.1% per year, 95%CI:-2.5, 0.4, p=0.149). Trends in England were relatively stable until 2005 onwards where there were significant decreasing trends for both alcohol-related (-6.2%, 95%CI:-8.98,-3.94, p<.001) and overall injury rates (-3.1%, 95%CI:-4.11,-2.16, p<.001), in which alcohol-related injuries had a stronger decline during this period.

Age comparisons indicated that in WA there was an increase in alcohol-related injuries in the 16-17 year age group, an increase of 2.8% (95%CI:1.9, 3.6, p<0.001) per year (from 14.3 to 22 per 10,000). In the 13-15 year age group there was a non-significant increase of 1.2% (95%CI:-0.2, 2.5, p=0.090) per year (from 4 to 4.8 per 10,000). This is in contrast to England where rates for the 16-17 year age group had a non-significant increase of 1.4% (-0.3, 3.1, p=0.101) per year and the rate for 13-15 year olds decreased by 3.7% (-5.4, -1.9, p<0.001) per year.

Gender comparisons showed that in WA boys aged 16-17 years had the highest rate of alcohol-related injury admissions at 27 per 10,000 in 2009, while the largest change in trends was for girls across both the 13-15 and 16-17 age groups (Figure 1). The rates of alcohol-related injury admissions in the 13-15 year age group increased by 2.8% (95%CI:0.9, 4.6, p=0.003) per year for girls and remained stable for boys (-0.4% per year, 95%CI:-1.9, 1.1, p=0.603). In the 16-17 year age group, girls also had a larger change in trends at 3.3% (95%CI:2.1, 4.5, p<0.001) per year compared to boys at 2.5% (95%CI:1.4, 3.5, p<0.001).

For peer review only - http://bmjopen.bmj.com/site/about/guidelines.xhtml

For peer review only

In England, there has been an overall increase of 4.1% (95%CI:2.3, 6.0, p<0.001) per year in alcohol-related injury rates for girls aged 16-17 (from 9 to 12 per 10,000) whereas rates have declined for all other groups. For boys aged 13-15 there has been a significant decrease of 6.5% (95%CI:-8.0, -5.0, p<0.001) per year. For girls aged 13-15 and boys aged 16-17 there have been non-significant decreases of 1.6% (95%CI:-3.7, 0.6, p=0.147) and 0.8% (95%CI:-2.5, 0.9, p=0.345) per year respectively. Alcohol-related injury rates in England have been decreasing for both genders since 2007, with the most recent rate in 2012 being much lower in England compared to Western Australia for boys aged 16-17 years.

In Western Australia, the predominant cause for alcohol-related injuries for 13-17 year old boys was unintentional, closely followed by intentional injuries. The opposite was true for 13-17 year old girls, where intentional injury rates were twice as high as unintentional injury rates. This difference was most notable among 16-17 year old girls where there were 11.5 per 10,000 intentional alcohol-related injuries in 2009 compared to 4.8 per 10,000 unintentional alcohol-related injuries (Figure 2). In England, the predominant cause of alcohol-related injury was intentional, with 16-17 year old girls in particular showing the highest rates (10.6 per 10,000 in 2009). This was one of the few trends that mirrored the pattern shown in Western Australia.

Alcohol-related injuries that were recorded as intentional are shown according to whether records indicated violence (which were predominantly assaults) or self-harm (Figure 3). In Western Australia, alcohol-related injuries caused by violence increased for both

For peer review only - http://bmjopen.bmj.com/site/about/guidelines.xhtml

For peer review only

genders. This is in contrast to England where violence related injury rates remained relatively stable. In WA violence-related injuries for boys aged 13-17 year old (4 per 10,000 in 2009) were the most prevalent intentional injuries, with the highest being for 16-17 year old boys at 8 per 10,000, increasing 4.8% (95%CI:2.8, 6.7, $p<0.001$) per year. For girls 16-17 year olds have higher rates (2.7 per 10,000 in 2009) compared to 13-15 year old girls, however they remained stable from 1990-2009 but increased significantly for 13-15 year olds by 7.1% per year (Figure 3). Self-harm predominated among girls in both WA and England, showing increasing rates in both countries, but a decline in England since 2007.

Unintentional alcohol-related injury rates have also increased in Western Australia for both boys and girls between 1990 and 2009 apart from 13-15 year old boys which remained stable. In England, unintentional alcohol-related injury rates have generally fallen or remained stable across both genders.

Absolute rates of alcohol-related injuries are shown for both countries for 2009, the most recent year in which comparable data was available for Western Australia (Table 1). For 13-17 year olds overall, Western Australia had higher rates of alcohol-related injuries in all categories other than self-harm. In both countries, 16-17 year olds had the highest rate of alcohol-related injuries, however the rate was twice as high among 16-17 year old boys in Western Australia (27.1/10,000) than in England (13.2/10,000). This pattern was consistent across both intentional and unintentional injuries. England had higher rates of alcohol-related self-harm, particularly among girls aged 13-15 (5.1/10,000) than Western Australia (2.5/10,000).

For peer review only - http://bmjopen.bmj.com/site/about/guidelines.xhtml

Table 1. Alcohol-related injury admission rates for children in Western Australia and England aged 13-17 years, 2009

Age group	Alcohol-related cause of injury	Western Australia			England		
		Boys Absolute rate 2009 (per 10,000)	Girls Absolute rate 2009 (per 10,000)	Total Absolute rate 2009 (per 10,000)	Boys Absolute rate 2009 (per 10,000)	Girls Absolute rate 2009 (per 10,000)	Total Absolute rate 2009 (per 10,000)
13-15 years	Injury	3.7 (2.3, 5.9)	6.0 (4.1, 8.8)	4.8 (3.6, 6.5)	4.6 (4.2, 5.1)	8.7 (8.1, 9.3)	6.6 (6.3, 7.0)
	Intentional	1.5 (0.7, 3.2)	3.7 (2.3, 6.0)	2.6 (1.7, 3.9)	1.8 (1.5, 2.1)	5.5 (5.0, 6.0)	3.6 (3.3, 3.9)
	Violence	0.9 (0.3, 2.3)	1.1 (0.5, 2.8)	1.0 (0.5, 1.9)	0.7 (0.5, 0.9)	0.5 (0.4, 0.7)	0.6 (0.5, 0.7)
	Self-harm	0.6 (0.2, 2.0)	2.5 (1.4, 4.6)	1.6 (0.9, 2.6)	1.1 (1.0, 1.4)	5.1 (4.7, 5.6)	3.1 (2.8, 3.3)
	Unintentional	2.2 (1.2, 4.0)	1.6 (0.8, 3.4)	1.9 (1.2, 3.0)	2.8 (2.5, 3.2)	3.6 (3.2, 4.0)	3.2 (2.9, 3.5)
16-17 years	Injury	27.1 (21.9, 33.5)	16.6 (12.6, 22.0)	22.0 (18.6, 26.1)	13.2 (12.4, 14.1)	14.1 (13.2, 15.0)	13.6 (13.0, 14.3)
	Intentional	12.9 (9.5, 17.6)	11.5 (8.3, 16.2)	12.2 (9.7, 15.4)	6.9 (6.3, 7.6)	10.6 (9.8, 11.5)	8.7 (8.2, 9.2)
	Violence	8.4 (5.7, 12.3)	2.7 (1.4, 5.4)	5.6 (4.0, 7.9)	2.9 (2.5, 3.3)	0.5 (0.4, 0.7)	1.7 (1.5, 2.0)
	Self-harm	4.8 (2.9, 8.0)	8.8 (6.0, 13.0)	6.8 (5.0, 9.2)	4.1 (3.7, 4.6)	10.2 (9.4, 11.0)	7.1 (6.6, 7.5)
	Unintentional	12.9 (9.5, 17.6)	4.8 (2.8, 8.0)	8.9 (6.8, 11.7)	6.5 (5.9, 7.1)	3.9 (3.4, 4.4)	5.2 (4.8, 5.6)

For peer review only - http://bmjopen.bmj.com/site/about/guidelines.xhtml

For peer review only

13-17 years	Injury	13.1 (10.8, 15.9)	10.3 (8.2, 12.9)	11.7 (10.1, 13.6)	8.2 (7.8, 8.7)	10.9 (10.4, 11.5)	9.5 (9.2, 9.9)
	Intentional	6.1 (4.6, 8.1)	6.9 (5.2, 9.0)	6.5 (5.3, 7.9)	3.9 (3.6, 4.2)	7.6 (7.2, 8.0)	5.7 (5.4, 6.0)
	Violence	3.9 (2.7, 5.6)	1.8 (1.0, 3.1)	2.9 (2.1, 3.9)	1.6 (1.4, 1.8)	0.5 (0.4, 0.6)	1.1 (1.0, 1.2)
	Self-harm	2.3 (1.5, 3.7)	5.1 (3.7, 7.0)	3.7 (2.8, 4.8)	2.4 (2.2, 2.6)	7.2 (6.8, 7.7)	4.7 (4.5, 5.0)
	Unintentional	6.5 (4.9, 8.5)	2.9 (1.9, 4.4)	4.7 (3.7, 6.0)	4.3 (4.0, 4.7)	3.7 (3.4, 4.0)	4.0 (3.8, 4.2)

For peer review only

For peer review only - http://bmjopen.bmj.com/site/about/guidelines.xhtml

Further analysis of the alcohol-related injury admission was conducted for Western Australia. Overall, the average length of stay associated with alcohol-related admissions was 2.6 days. We found that of self-harm injuries in boys, 24% were alcohol-related, compared to 13% of self-harm injuries in girls. For both genders, 15-17% of assault injuries were alcohol-related, compared to only 3% of accidental injuries. For 16-17 year olds alcohol was associated with 18% of assault-related injuries compared to 12% in 13-15 year olds. In 2009 this was even higher at 27% of assault-related injuries being associated with alcohol in the 16-17 year old age group. The predominant type of alcohol-related assault was assault by bodily force at (53%), followed by assault by unspecified means (18%), assault by blunt object (14%) and assault by sharp object (10%). In 3% of alcohol-related assault admissions the assault was rape.

DISCUSSION

Despite the decline in hospital admissions in Western Australia from 1990 to 2009 for injury for adolescents aged 13-17 there has been an increase in alcohol-related injuries by 2.3% per year. This increase has been largest in the 16-17 year old age group with boys aged 16-17 years having the highest rates. However girls in both the 13-15 and 16-17 age groups had the greatest increase in trends of 2.7% and 3.2% per year respectively. This substantiates and builds on a previous Australian study showing increases in alcohol-related injury admissions in 16-17 year olds till 2005 and a large rise in hospital presentations for girls.[4]

For peer review only - http://bmjopen.bmj.com/site/about/guidelines.xhtml

In contrast to the increasing rates of alcohol-related injuries in Western Australia, England's rates remained relatively stable over the study period. Most age and gender groups have shown decreasing or stable rates of alcohol-related injuries since 2007, with the exception of 16-17 year old girls. This suggests that alcohol-related harm in adolescents is a problem that can be reduced. Although coding practices can affect the magnitude of the trend, a decrease in overall alcohol-related harms was found despite an increase in England's use of secondary diagnosis codes[18], and is consistent with surveys of 11-15 year olds from 2001-2011 showing a decrease in frequent drinking.[19] In addition, England's Health Behaviour in School-aged Children Report found from 2002-2014 there was a 23-26% reduction in the proportion of 15 year olds reporting drinking to excess.[20] Recent data from Public Health England has also found a 13.9% fall in hospital admissions caused by alcohol in England for under 18's which corroborates our findings. [21]

In England, self-harm was the leading cause of alcohol-related injury particularly among girls, an issue which warrants further attention. Self-harm was also the most common form of intentional alcohol-related injury for girls in Western Australia. Research has found many adolescents admitted for injuries with self-harm also had drug or alcohol misuse coded and increased likelihood of having experienced violence, suggesting a need for strategies that address multiple forms of adversity. [22]

A large minority of injuries in Western Australia were unintentional injuries however there was a rise in violence-related admissions. Overall alcohol was associated with 16% of assault-related admissions and this was higher in 2009 for adolescents aged 16-17

For peer review only - http://bmjopen.bmj.com/site/about/guidelines.xhtml

years, with 20% of assaults associated with alcohol for girls and 30% for boys. This highlights the growing issue in Australia with the rise in violent injuries related to alcohol and the associated harm that adolescents are experiencing. It is important that this issue be monitored, firstly so we know the extent of harm and the burden on the health system of alcohol-related injuries and secondly that as strategies are implemented to reduce this burden we can look at the impact on the rate of admissions.

Unfortunately our data only captures when the individual admitted for the injury has an alcohol-related diagnosis not when the perpetrator had been affected by alcohol. Therefore this would certainly underestimate the rates of injury associated with alcohol use which is also important in capturing the burden. Research suggests alcohol use by the perpetrator was a factor in over half of physical and sexual assaults,[23] and has established associations with child maltreatment [24 25] and road traffic crashes.[26] Future research could use data from police and corrective services to investigate alcohol-related harm. Also our data only relates to injuries requiring hospitalisation not those seen in the Emergency Departments (ED) therefore our injury admissions would relate to more serious injuries and violence. Another limitation is that changes in coding over time may influence apparent trends. Our study shows that at least in WA there is an increase in recognition (whether due to more rigorous reporting or as a rise in actual incidents) of the influence of alcohol in injury admissions.

Using hospital admissions data allows monitoring of trends over time and country comparisons can generate hypotheses about policies or service factors that may account for differences. This is the first study examining and comparing trends in alcohol-related

For peer review only - http://bmjopen.bmj.com/site/about/guidelines.xhtml

harm in adolescents in Western Australia and England. Identification of age and gender groups at greatest risk, and the types of harm they experience is necessary to inform public health initiatives targeting alcohol-related harm in adolescents. In England it is unclear which specific policies or practices have influenced the decline, but a strategy for reducing alcohol-related harm has been in place since 2004. An annual tax escalator was implemented to boost duties on all alcohol[27] in 2008 and Challenge 25 was subsequently introduced, requiring identification from people who look less 25 years old when purchasing alcohol. Researchers have also stated that the economic downturn in England and the rise in alcohol taxation may have contributed to stemming the rise in alcohol-related deaths[28]. English researchers have also noted falls in other adversity-related admissions from 2005-11 and discussed the implementation of programs (eg. Sure Start, Alcohol Misuse Enforcement Campaign) which may have influenced rates[29].

Some strategies and programs are in place in Australia to address youth drinking and alcohol-related harm.[2 3] These appear to be having some positive effects, with adolescents being the only group that has shown a decrease in the proportion of people who drink at risky levels.[3] Nonetheless, our research shows that rates of alcohol-related injury admissions increased in Western Australia, consistent with findings for Victoria, Australia.[4] Possible explanations for this discordance include the hypothesis that sub-groups excluded from surveys (such as homeless or truant youth, those who left school prior to Year 12 for school surveys, or those that chose not to participate) have had increased drinking or that sub-groups have increased excessive and risky drinking.[30]

Australia and England follow a number of the World Health Organisation recommendations for reducing alcohol-related harm including maximum blood alcohol

For peer review only - http://bmjopen.bmj.com/site/about/guidelines.xhtml

levels while driving, and restrictions on age, sales to intoxicated people, and promotion of alcoholic beverages at activities targeting young people.[11] However more clearly needs to be done to reduce the rising rates of alcohol-related harm in Western Australia and in England despite the declining rates Professor Kevin Fenton, Director of Health and Wellbeing at Public Health England has stated that ‘while this is promising… current levels of harm caused by alcohol remain unacceptably high, especially within the most deprived communities, who suffer the most from poor health in general.’[31]

Evidence suggests the optimal package of interventions also includes measures to reduce the affordability and availability of alcohol.[32-34] There is evidence that pricing policy can significantly reduce alcohol consumption [35 36] and alcohol-related hospital admissions [34] and deaths [37]. Minimum unit pricing or volumetric pricing are considered effective and cost-effective solutions.[38] In 2012, Scotland followed Canada in legislating for minimum alcohol unit pricing.[39] Recent research has found the majority of young risky drinkers support evidence-based policies including price increases if revenue is used to support treatment and prevention of alcohol problems.[40]

For peer review only - http://bmjopen.bmj.com/site/about/guidelines.xhtml

CONTRIBUTORSHIP STATEMENT

All authors have contributed to the conceptualisation of this paper and/or to the writing of this paper and/or contributed to the analysis of data and its interpretation.

COMPETING INTERESTS

The authors have no competing interests.

FUNDING AND ACKNOWLEDGEMENTS

The Policy Research Unit in the Health of Children, Young People and Families (CPRU), which is funded by the England Department of Health Policy Research Programme. This is an independent report commissioned and funded by the Department of Health. We thank members of CPRU: Terence Stephenson, Catherine Law, Amanda Edwards, Steve Morris, Helen Roberts, Cathy Street, Russell Viner and Miranda Wolpert. RG and AGI were also partly supported by awards establishing the Farr Institute of Health Informatics Research (MR/K006584/1). Melissa O'Donnell was supported by a National Health and Medical Research Council Early Career Fellowship [1012439]. This research was also supported by an Australian Research Council Linkage Project Grant [LP100200507] and an Australian Research Council Discovery Grant [DP110100967]. We also acknowledge the partnership of the Western Australian Government Departments of Health, Child Protection, Education, Disability Services, Corrective Services and the Attorney General, which provided data for this project. The views expressed are not necessarily those of the Government Departments that have commissioned or supported this project.

DATA SHARING STATEMENT

The data utilised in this paper is owned by our respective government departments and therefore would require permissions by these departments for others to access.

For peer review only - http://bmjopen.bmj.com/site/about/guidelines.xhtml

REFERENCES

1. Degenhardt L, Stockings E, Patton G, et al. The increasing global health priority of substance use in young people. The Lancet Psychiatry 2016;**3**(3):251-64.
2. Moodie R, Daube M, Carnell K, et al. Australia: The healthiest country by 2020 National Preventative Health Strategy - the roadmap for action. Canberra Australian Government Preventative Health Taskforce, 2009.
3. Moodie R, Daube M, Zimmet P, et al. Taking Preventative Action: A Response to Australia: The Healthiest Country by 2020 - The Report of the National Preventative Health Task Force. Canberra: Australian Government Preventative Health Taskforce, 2010.
4. Livingston M. Recent trends in risky alcohol consumption and related harm among young people in Victoria, Australia. Australian and New Zealand Journal of Public Health 2008;**32**(3):266-71.
5. Morgan A, McAtamney A. Key issues in alcohol-related violence. Research in Practice: Summary no. 4. . Canberra: Australian Institute of Criminology, 2009.
6. Plant M, Plant M, Thornton C. People and places: Some factors in the alcohol-violence link. Journal of Substance Use 2002;**7**(4):207-13.
7. Krug EG, Dahlberg LL, Mercy JA, et al. World report on violence and health. Geneva: World Health Organization, 2002.
8. World Health Organisation. Global strategy to reduce the harmful use of alcohol. Geneva: WHO, 2010.
9. Morgan A, McAtamney A. Key issues in alcohol-related violence. Research in Practice: Summary no 4. Canberra: Australian Institute of Criminology, 2009.
10. Gonzalez-Izquierdo A, Ward A, O'Donnell M, et al. Cross-country comparison of victimisation-related injury admission in children and adolescents in England and Western Australia. BMC Health Services Research 2013;**13**(1):260.
11. WHO. Global status report on alcohol and health 2014 Geneva: World Health Organization, 2014.
12. Toumbourou JW, Stockwell T, Neighbors C, et al. Adolescent Health 4: Interventions to reduce harm associated with adolescent substance use. Lancet 2007;**369**(9570):1391-401.
13. Egerton-Warburton D, Gosbell A, Wadsworth A, et al. Survey of alcohol-related presentations to Australasian emergency departments. Medical Journal of Australia 2014;**201**(10):584-87.
14. Gonzalez-Izquierdo A, Ward A, O'Donnell M, et al. Cross-country comparison of victimisation-related injury admission in children and adolescents in England and Western Australia. BMC Health Services Research 2013;**13**:260.
15. NHS. The information centre for health and social care. HES online. The approvals process. 2012. 6-8-2012.
16. Kuhn L, Davidson LL, Durkin MS. Use of poisson regression and time series analysis for detecting changes over time in rates of child injury following a prevention program. American Journal of Epidemiology 1994;**140**(10):943-55.
17. SAS Version 9.1 [program]. Cary NC: SAS Institute Inc., 2004.
18. Lifestyle Statistics Health and Social Care Information Centre. Statistics on Alcohol: England, 2013. England: UK Government, 2013.
19. Fuller E, Gill V, Hawkins V, et al. Smoking, drinking and drug use among young people in England in 2011, NatCen Social Research. London, 2012.

For peer review only

For peer review only - http://bmjopen.bmj.com/site/about/guidelines.xhtml

20. Brooks F, Magnusson J, Klemera E, et al. HBSC England National Report 2014. Hatfield, UK: University of Hertfordshire, 2015.
21. Public Health England. Local Alcohol Profiles for England, June 2015. Statistical Bulletin. London: Public Health England, 2015.
22. Herbert A, Gilbert R, González-Izquierdo A, et al. Violence, self-harm and drug or alcohol misuse in adolescents admitted to hospitals in England for injury: a retrospective cohort study. BMJ Open 2015;**5**(2):e006079.
23. Connor J, You R, Casswell S. Alcohol-related harm to others: a survey of physical and sexual assault in New Zealand. New Zealand Medical Journal 2009;**122**(1303).
24. Gilbert R, Widom CS, Browne K, et al. Burden and consequences of child maltreatment in high-income countries. Lancet 2009;**373**(9657):68-81.
25. Laslett A-M, Ferris J, Dietze P, et al. Social demography of alcohol-related harm to children in Australia. Addiction 2012;**107**(6):1082-89.
26. Bramwell J, Hill D, Thompson PE. Reported road crashes in Western Australia 2012. Perth: Road Safety Council of Western Australia, 2014.
27. Gilmore I, Anderson W, Bauld L, et al. Health first: An evidence-based alcohol strategy for the UK Stirling: University of Stirling, 2013.
28. Sheron N, Gilmore I. Effect of policy, econmics, and the changing alcohol marketplace on alcohol related deaths in England and Wales. British Medical Journal 2016;**353**:i1860
29. Herbert A, Gonzalez-Izquierdo A, McGhee J, et al. Time-trends in rates of hospital admissions of adolescents for violent, self-inflicted or drug/alcohol-relted injury in England and Scotland, 2005-11: population-based analysis. Journal of Public Health 2016;doi:10.1093/pubmed/fdw020.
30. Livingston M, Matthews S, Barratt MJ, et al. Diverging trends in alcohol consumption and alcohol-related harm in Victoria. Australian and New Zealand Journal of Public Health 2010;**34**(4):368-73.
31. Public Health England. Alcohol-related hospital admissions decrease among under 18s. London: Public Health England, 2015.
32. Anderson P, Chisholm D, Fuhr DC. Alcohol and global health 2: Effectiveness and cost-effectiveness of policies and programmes to reduce the harm caused by alcohol. Lancet 2009;**373**(9682):2234-46.
33. Casswell S, Thamarangsi T. Alcohol and global health 3: Reducing harm from alcohol: call to action. Lancet 2009;**373**(9682):2247-57.
34. Stockwell T, Zhao J, Martin G, et al. Minimum alcohol prices and outlet densities in British Columbia, Canada: Estimated impacts on alcohol-attributable hospital admissions. American Journal of Public Health 2013;**103**(11):2014-20.
35. Wagenaar AC, Salois MJ, Komro KA. Effects of beverage alcohol price and tax levels on drinking: a meta-analysis of 1003 estimates from 112 studies. Addiction 2009;**104**(2):179-90.
36. Stockwell T, Zhao J, Giesbrecht N, et al. The raising of minimum alcohol prices in Saskatchewan, Canada: Impacts on consumption and implications for public health. American Journal of Public Health 2012;**102**(12):e103-e10.
37. Chikritzhs T, Stockwell T, Pascal R. The impact of the Northern Territory's Living With Alcohol program, 1992–2002: revisiting the evaluation. Addiction 2005;**100**(11):1625-36.

For peer review only - http://bmjopen.bmj.com/site/about/guidelines.xhtml

38. Sharma A, Vandenberg B, Hollingsworth B. Minimum pricing of alcohol versus volumetric taxation: Which policy will reduce heavy consumption without adversely affecting light and moderate consumers? PLoS ONE 2014;**9**(1):1-13.
39. Leicester A. Alcohol pricing and taxation policies, IFS Briefing Note BN124: Institute for Fiscal Studies, 2011.
40. Lam T, Lenton S, Burns L, et al. Alcohol policy impact on young risky drinkers and their support for proposed measures. Australian and New Zealand Journal of Public Health 2015;**39**(2):12-134.

For peer review only

For peer review only - http://bmjopen.bmj.com/site/about/guidelines.xhtml

Figure 1. Rate of hospital admissions for alcohol-related injury in Western Australia and England (per 10,000 persons) for adolescents, aged 13-15 and 16-17 years. Light solid= Alcohol-related injury (13-15yrs); dark solid=Alcohol-related injury (16-17yrs)

See Supplementary file for Figure 1

Figure II. Rate of intentional and unintentional alcohol-related injury hospital admissions in Western Australia and England (per 10,000 persons) for adolescents aged 13-15 and 16-17years. Light dash=intentional (13-15yrs); Light solid=unintentional (13-15yrs); dark dash=intentional (16-17yrs); dark solid=unintentional (16-17yrs).

See Supplementary file for Figure 2

Figure III. Rate of intentional alcohol-related injury hospital admissions in Western Australia and England (per 10,000 persons) for adolescents aged 13-17 years, by violence-related and self-harm admissions. Dark dash=violence related (13-17yrs); dark solid=self-harm (13-17yrs).

See Supplementary file for Figure 3.

For peer review only

For peer review only - http://bmjopen.bmj.com/site/about/guidelines.xhtml

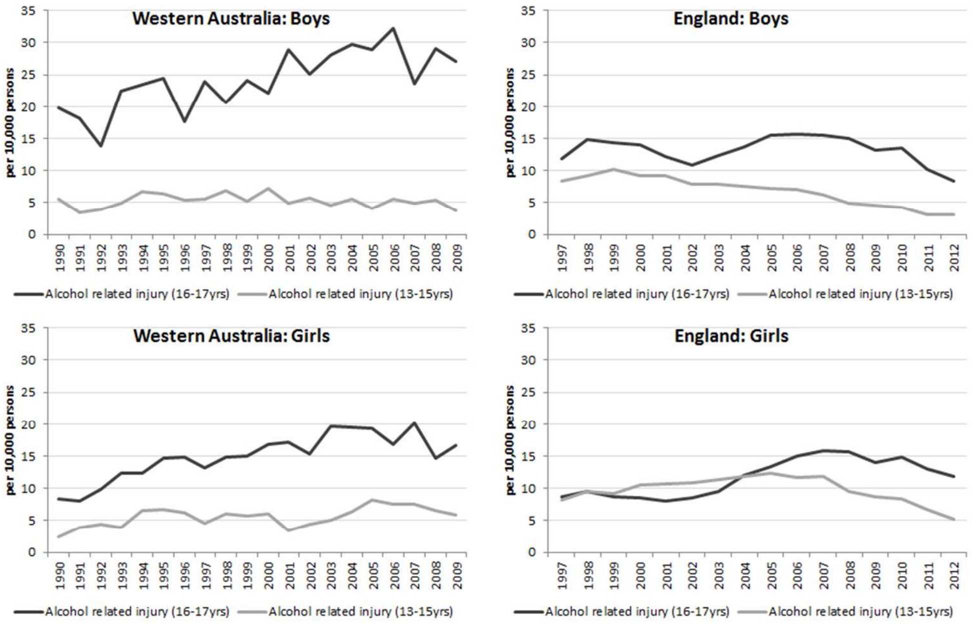

Figure 1. Rate of hospital admissions for alcohol-related injury in Western Australia and England (per 10,000 persons) for adolescents, aged 13-15 and 16-17 years. Light solid= Alcohol-related injury (13-15yrs); dark solid=Alcohol-related injury (16-17yrs)

80x51mm (300 x 300 DPI)

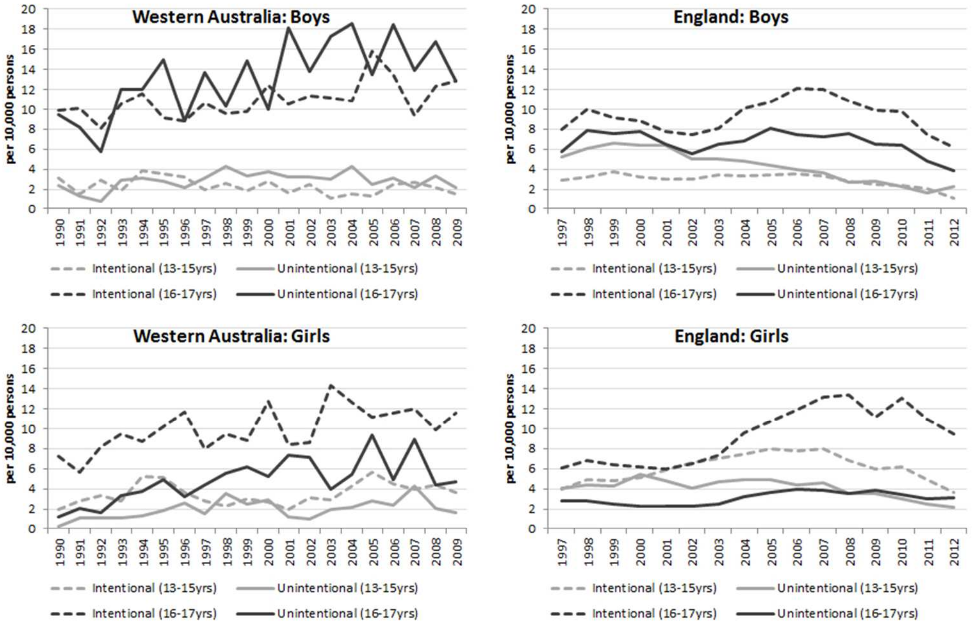

Figure II. Rate of intentional and unintentional alcohol-related injury hospital admissions in Western Australia and England (per 10,000 persons) for adolescents aged 13-15 and 16-17years. Light dash=intentional (13-15yrs); Light solid=unintentional (13-15yrs); dark dash=intentional (16-17yrs); dark solid=unintentional (16-17yrs).

80x51mm (300 x 300 DPI)

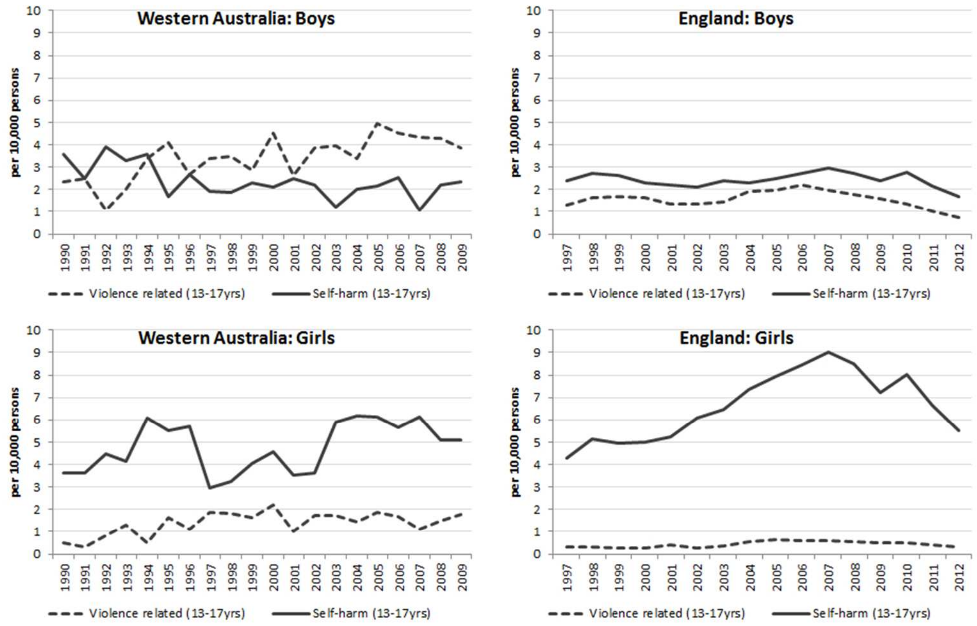

Figure III. Rate of intentional alcohol-related injury hospital admissions in Western Australia and England (per 10,000 persons) for adolescents aged 13-17 years, by violence-related and self-harm admissions. Dark dash=violence related (13-17yrs); dark solid=self-harm (13-17yrs).

80x51mm (300 x 300 DPI)

STROBE Statement—checklist of items that should be included in reports of observational studies

	Item No	Recommendation
**Title and abstract**	A - Title<br>B - Abstract	(*a*) Indicate the study's design with a commonly used term in the title or the abstract
		(*b*) Provide in the abstract an informative and balanced summary of what was done and what was found
**Introduction**		
Background/rationale	Pg 5 and 6	Explain the scientific background and rationale for the investigation being reported
Objectives	Pg 6	State specific objectives, including any prespecified hypotheses
**Methods**		
Study design	Pg 6 and 8	Present key elements of study design early in the paper
Setting	Pg 6	Describe the setting, locations, and relevant dates, including periods of recruitment, exposure, follow-up, and data collection
Participants	A- Pg6 and 7 population based cohort so not necessary to discuss follow-up	(*a*) *Cohort study*—Give the eligibility criteria, and the sources and methods of selection of participants. Describe methods of follow-up<br>*Case-control study*—Give the eligibility criteria, and the sources and methods of case ascertainment and control selection. Give the rationale for the choice of cases and controls<br>*Cross-sectional study*—Give the eligibility criteria, and the sources and methods of selection of participants
		(*b*) *Cohort study*—For matched studies, give matching criteria and number of exposed and unexposed<br>*Case-control study*—For matched studies, give matching criteria and the number of controls per case
Variables	Pg 6 and 7	Clearly define all outcomes, exposures, predictors, potential confounders, and effect modifiers. Give diagnostic criteria, if applicable
Data sources/ measurement	Pg 6 and 7	For each variable of interest, give sources of data and details of methods of assessment (measurement). Describe comparability of assessment methods if there is more than one group
Bias	None (discussed in limitations – eg coding changes)	Describe any efforts to address potential sources of bias
Study size	Not Applicable Population based cohort study	Explain how the study size was arrived at
Quantitative variables	Pg 8	Explain how quantitative variables were handled in the analyses. If applicable, describe which groupings were chosen and why
Statistical methods	A and B – Pg 8<br>C – None<br>D- Not applicable<br>E - None	(*a*) Describe all statistical methods, including those used to control for confounding
		(*b*) Describe any methods used to examine subgroups and interactions
		(*c*) Explain how missing data were addressed
		(*d*) *Cohort study*—If applicable, explain how loss to follow-up was addressed<br>*Case-control study*—If applicable, explain how matching of cases and controls was addressed

For peer review only - http://bmjopen.bmj.com/site/about/guidelines.xhtml

	Cross-sectional study—If applicable, describe analytical methods taking account of sampling strategy
	(*e*) Describe any sensitivity analyses

For peer review only

For peer review only - http://bmjopen.bmj.com/site/about/guidelines.xhtml

**Results**		
Participants	Pg 6- Population based cohort study	(a) Report numbers of individuals at each stage of study—eg numbers potentially eligible, examined for eligibility, confirmed eligible, included in the study, completing follow-up, and analysed
		(b) Give reasons for non-participation at each stage
		(c) Consider use of a flow diagram
Descriptive data	A - Pg 6<br>B – Not applicable<br>C – Not applicable	(a) Give characteristics of study participants (eg demographic, clinical, social) and information on exposures and potential confounders
		(b) Indicate number of participants with missing data for each variable of interest
		(c) *Cohort study*—Summarise follow-up time (eg, average and total amount)
Outcome data	P9-15	*Cohort study*—Report numbers of outcome events or summary measures over time
		Case-control study—Report numbers in each exposure category, or summary measures of exposure
		Cross-sectional study—Report numbers of outcome events or summary measures
Main results	Pg 9-15	(*a*) Give unadjusted estimates and, if applicable, confounder-adjusted estimates and their precision (eg, 95% confidence interval). Make clear which confounders were adjusted for and why they were included
		(*b*) Report category boundaries when continuous variables were categorized
		(*c*) If relevant, consider translating estimates of relative risk into absolute risk for a meaningful time period
Other analyses	None	Report other analyses done—eg analyses of subgroups and interactions, and sensitivity analyses
**Discussion**		
Key results	Pg 15-16	Summarise key results with reference to study objectives
Limitations	Pg 17	Discuss limitations of the study, taking into account sources of potential bias or imprecision. Discuss both direction and magnitude of any potential bias
Interpretation	Pg 15-19	Give a cautious overall interpretation of results considering objectives, limitations, multiplicity of analyses, results from similar studies, and other relevant evidence
Generalisability	Pg 17	Discuss the generalisability (external validity) of the study results
**Other information**		
Funding	Pg 20	Give the source of funding and the role of the funders for the present study and, if applicable, for the original study on which the present article is based

*Give information separately for cases and controls in case-control studies and, if applicable, for exposed and unexposed groups in cohort and cross-sectional studies.

Note: An Explanation and Elaboration article discusses each checklist item and gives methodological background and published examples of transparent reporting. The STROBE checklist is best used in conjunction with this article (freely available on the Web sites of PLoS Medicine at http://www.plosmedicine.org/, Annals of Internal Medicine at http://www.annals.org/, and Epidemiology at http://www.epidem.com/). Information on the STROBE Initiative is available at www.strobe-statement.org.

For peer review only

BMJ Open

Trends in alcohol-related injury admissions in adolescents in Western Australia and England: Population-based cohort study

Journal:	*BMJ Open*
Manuscript ID	bmjopen-2016-014913.R2
Article Type:	Research
Date Submitted by the Author:	23-Mar-2017

Complete List of Authors:	O'Donnell, Melissa; University of Western Australia, Telethon Kids Institute<br>Sims, Scott; Telethon Kids Institute, University of Wester Australia,<br>Maclean, Miriam; Telethon Kids Institute, University of Western Australia,<br>Gonzalez-Izquierdo, Arturo; University College London<br>Gilbert, Ruth; University College London, Institute of Child Health<br>Stanley, Fiona; University of Western Australia, Telethon Kids Institute

**Primary Subject Heading**:	Public health
Secondary Subject Heading:	Epidemiology, Public health
Keywords:	alcohol, adolescents, EPIDEMIOLOGY, Non-accidental injury < PAEDIATRICS

SCHOLARONE™ Manuscripts

For peer review only

Running Head: TRENDS IN ALCOHOL-RELATED INJURY ADMISSIONS

Title: Trends in alcohol-related injury admissions in adolescents in Western Australia and England: Population-based cohort study

Melissa O'Donnell, BPsych (Hons), MPsych, GradDip Ed, PhD, NHMRC Postdoctoral Research Fellow, Telethon Kids Institute, University of Western Australia, Perth, Australia. Email: melissao@ichr.uwa.edu.au

Scott Sims, BSc, MBiostat, Data Analyst, Telethon Kids Institute, University of Western Australia, Perth, Australia. Email: Scott.Sims@telethonkids.org.au

Miriam J Maclean, BPsych (Hons), MSc, Telethon Kids Institute, University of Western Australia, Perth, Australia. Email: Miriam.Maclean@telethonkids.org.au

Arturo Gonzalez-Izquierdo, PhD, Data Scientist, Farr Institute of Health Informatics Research, University College London, London. Email: arturo.gonzalez-izquierdo@ucl.ac.uk

Ruth Gilbert, MD, MSc, Professor of Clinical Epidemiology, Population Policy and Practice Programme, University College London, Institute of Child Health, London. Email: r.gilbert@ucl.ac.uk

For peer review only

Fiona J Stanley, MD, MSc, FFPHM, FAFPHM, Research Professor, Telethon Kids Institute, University of Western Australia, Perth, Australia. Email: Fiona.Stanley@telethonkids.org.au

Corresponding Author and Address: Melissa O'Donnell, Telethon Kids Institute, University of Western Australia, 100 Roberts Road, Subiaco, Western Australia. Telephone: (08) 9489 7778. Email: Melissa.O'Donnell@telethonkids.org.au

Keywords: Injury rates, trends, alcohol, adolescents, youth

Number of Tables=1

Number of Figures=3

Word Count=3110

For peer review only

For peer review only - http://bmjopen.bmj.com/site/about/guidelines.xhtml

Abstract

Background: Alcohol-related harm in young people is now a global health priority. We examined trends in hospital admissions for alcohol-related injuries for adolescents in Western Australia (WA) and in England, identified groups most at risk and determined causes of injuries.

Methods: Annual incidence rates for alcohol-related injury rates were calculated using population-level hospital admissions data for WA and England. We compared trends in different types of alcohol-related injury by age and gender.

Results: Despite a decrease in the overall rate of injury admissions for 13-17 year olds in WA, alcohol-related injuries have increased significantly from 1990-2009 (from 8 to 12 per 10,000). Conversely, alcohol-related injury rates have declined in England since 2007. In England self-harm is the most frequently recorded cause of alcohol-related injury. In WA, unintentional injury is most common, however violence-related harm is increasing for boys and girls.

Conclusions: Alcohol-related harm of sufficient severity to require hospital admission is increasing among adolescents in Western Australia. Declining trends in England suggests this trend is not inevitable or irreversible. More needs to be done to address alcohol-related harm, and ongoing monitoring is required to assess the effectiveness of strategies.

Strengths and limitations of this study

- This study investigates trends in adolescent hospital admissions for injuries and the documented causes of these injuries.
- While trends in alcohol-related admissions has been reported in previous research this adds to the knowledge of trends in different age groups and causes of injury.

For peer review only - http://bmjopen.bmj.com/site/about/guidelines.xhtml

- The limitations of this research is that only individuals admitted for injuries who have an alcohol-related diagnosis will be captured not when the perpetrator had been affected by alcohol therefore underestimating rates of injury associated with alcohol use.
- There also could be changes in coding practices which may contribute to changes in trends.

For peer review only

For peer review only - http://bmjopen.bmj.com/site/about/guidelines.xhtml

INTRODUCTION

Substance use in young people is considered a global health priority with young people highlighted as a priority group in the World Health Organisations Global strategy on Alcohol[1]. Concerns about the use of alcohol among young people have been rising over the last decade with young people the age group most likely to consume alcohol at levels of high risk. [2,3] Whilst recent Australian survey data has indicated that adolescents who are drinking at risky levels has declined this conflicts with increasing evidence of alcohol-related harm, particularly increases in alcohol-related hospital presentations by 16-17 year olds.[4] Alcohol-related violence is a growing problem among youth [5] and heavy drinking and intoxication is associated with violent offences.[6,7]

Given alcohol use at harmful levels is a significant risk factor for violence, strategies to reduce violence need to address alcohol misuse[8,9]. We have previously shown that violence-related injury accounts for almost a quarter of all injury admissions to hospital for adolescents aged 16 to 17 years.[10] Clinicians need information on the burden of alcohol-related injury admissions and the cause of those injuries in order to set in place services to address harmful drinking and reduce the risk of future injury or other health consequences. In addition, trends in alcohol-related injury admissions, and variation in trends between genders can inform policy about high risk groups. Variation in these patterns between countries contributes to evidence on potential service, policy, commercial or cultural influences on alcohol-related injury.

The aim of this study was to determine trends in alcohol-related injury admissions to hospital among adolescents in Western Australia and England and whether these

For peer review only - http://bmjopen.bmj.com/site/about/guidelines.xhtml

admissions are violence-related. Western Australia and England have similar health systems and have comparable rates of average daily alcohol consumption. [11] We also examined whether trends varied by gender and age-groups to determine groups most at risk. Our study focussed on adolescents aged 13-17 years as this is a priority age group for early intervention and prevention of harmful patterns of alcohol use.[12]

METHODS

Datasets

This study utilised hospital admissions data from England and Western Australia (WA). WA data included adolescents with injury admissions between 13 and 17 years of age to both private and public hospitals between 1990 and 2009. In England, we included the same age group for injury admissions to the National Health Service (NHS) in England between 1997 and 2012. Admissions were excluded if they were planned/scheduled admissions. Therefore admissions were included if patients were admitted through the hospitals admissions process for a period of care (for any length of time) due to a sudden health issue. In England each episode of care contains up to 20 diagnostic codes which contain external cause codes and in WA up to 21 diagnostic codes and 4 external cause codes. Both countries use International Classification of Diseases (ICD) coding systems with Western Australia moving from ICD-9 to ICD-10 on 1st July 1999, with England using only ICD-10 from 1997 onwards.

We defined injury admissions based on any diagnostic code for injury, poisoning or other consequences of external cause (ICD Codes: S or T). In WA ICD-10 and their equivalent

For peer review only - http://bmjopen.bmj.com/site/about/guidelines.xhtml

codes in ICD-9 were used. Alcohol-related injuries were identified if they contained ICD-10 diagnostic codes which have been used as identifiers for alcohol-related admissions in previous studies [4 13]: F10, E24.4, G31.2, G62.1, G72.1 I42.1, K29.2, K70, K85.2, K86.0, R78.0, T50.6, T51, X45, X65, Y15, Y91, Z50.2, Z71.4, Z72.1.

Causes of injury were classified as intentional if codes were recorded for assault, (ICD-10: Y04, Y05, X85-Y03, Y08-Y09) maltreatment (ICD-10: T74, T73, Y06, Y07), self-harm, (ICD-10: X60-X69, X70-X84, Z91.5), or undetermined cause (ICD-10: Y20-Y34, Z04.5, Z04.8). The sub-group of violence-related injuries was defined by codes related to assault, maltreatment, or undetermined cause which have been used in previous work[14]. Undetermined cause was included as they are an indication of potential assault and self-harm, however they only make up a small percent of our intentional injuries (<2%) and alcohol-related injuries (<1%). If individuals had codes in multiple categories (e.g. assault and self-harm) they were included in each category. Unintentional injuries were those injury admissions that contained codes of 'accident' as the external cause, including road traffic crashes (V01-V99, W00-X59).

Ethics

This study had ethics approval from the University of Western Australia Human Ethics Committee, the Department of Health Human Research Ethics Committee and the Western Australian Aboriginal Human Information and Ethics Committee. Use of anonymised data in England for the purpose in this study did not require research ethics approval but met the requirements of the data provider.[15]

For peer review only - http://bmjopen.bmj.com/site/about/guidelines.xhtml

Analyses

Annual incidence rates for maltreatment-related admissions were calculated by dividing the number of children who had these admissions by age-specific population estimates for each calendar year. In WA the denominator data was sourced from the Australian Bureau of Statistics population estimates (ABS, 2009). In England population denominators were based on mid-year population estimates obtained from the UK Office of National Statistics website (UK National Statistics, 2009). Time trends in the annual incidence of admissions were examined using Poisson regression and percentage change per year reported including confidence intervals to indicate significance. Poisson regression has been recommended for quantifying time trends of discrete events such as injury admissions[16]. All trends are presented as effect sizes with 95% confidence intervals (CI) and associated p-values and were considered statistically significant at the $p<0.05$ level if the CI did not include zero. A statistically non-significant trend ($p>0.05$) represents an inconclusive result. Due to some injury categories being relatively rare, there may be an effect which is undetected due to low sample sizes, thus it is still important to consider effect sizes and confidence intervals of trends despite non-significant p-values. We compared absolute rates in 2009 as this was the most recent year for data common to both countries. SAS release 9.1 was used to conduct all analyses.[17]

RESULTS

Injury admissions for any type of injury have declined in both WA and England over the last 10 to 20 years. However despite this decrease WA hospital admissions for alcohol-related injuries for 13-17 year olds increased during 1990-2009 from 8.2 to 12 per 10,000, an increase of 2.3% (95%CI:1.5, 3.1, $p<0.001$) per year. Conversely, in England,

For peer review only - http://bmjopen.bmj.com/site/about/guidelines.xhtml

the rate of admissions for alcohol-related injuries decreased marginally from 9.1 to 6.5 per 10,000 people, over the period of available data from 1997 to 2012, although this was a non-significant decrease (1.1% per year, 95%CI:-2.5, 0.4, p=0.149). Trends in England were relatively stable until 2005 onwards where there were significant decreasing trends for both alcohol-related (-6.2%, 95%CI:-8.98,-3.94, p<0.001) and overall injury rates (-3.1%, 95%CI:-4.11,-2.16, p<0.001), in which alcohol-related injuries had a stronger decline during this period.

Age comparisons indicated that in WA there was an increase in alcohol-related injuries in the 16-17 year age group, an increase of 2.8% (95%CI:1.9, 3.6, p<0.001) per year (from 14.3 to 22 per 10,000). In the 13-15 year age group there was a non-significant increase of 1.2% (95%CI:-0.2, 2.5, p=0.090) per year (from 4 to 4.8 per 10,000). This is in contrast to England where rates for the 16-17 year age group had a non-significant increase of 1.4% (-0.3, 3.1, p=0.101) per year and the rate for 13-15 year olds decreased by 3.7% (-5.4, -1.9, p<0.001) per year.

Gender comparisons showed that in WA boys aged 16-17 years had the highest rate of alcohol-related injury admissions at 27 per 10,000 in 2009, while the largest change in trends was for girls across both the 13-15 and 16-17 age groups (Figure 1). The rates of alcohol-related injury admissions in the 13-15 year age group increased by 2.8% (95%CI:0.9, 4.6, p<0.01) per year for girls and remained stable for boys (-0.4% per year, 95%CI:-1.9, 1.1, p=0.603). In the 16-17 year age group, girls also had a larger change in trends at 3.3% (95%CI:2.1, 4.5, p<0.001) per year compared to boys at 2.5% (95%CI:1.4, 3.5, p<0.001).

For peer review only - http://bmjopen.bmj.com/site/about/guidelines.xhtml

For peer review only

In England, there has been an overall increase of 4.1% (95%CI:2.3, 6.0, $p<0.001$) per year in alcohol-related injury rates for girls aged 16-17 (from 9 to 12 per 10,000) whereas rates have declined for all other groups. For boys aged 13-15 there has been a significant decrease of 6.5% (95%CI:-8.0, -5.0, $p<0.001$) per year. For girls aged 13-15 and boys aged 16-17 there have been non-significant decreases of 1.6% (95%CI:-3.7, 0.6, p=0.147) and 0.8% (95%CI:-2.5, 0.9, p=0.345) per year respectively. Alcohol-related injury rates in England have been decreasing for both genders since 2007, with the most recent rate in 2012 being much lower in England compared to Western Australia for boys aged 16-17 years.

In Western Australia, the predominant cause for alcohol-related injuries for 13-17 year old boys was unintentional, closely followed by intentional injuries. The opposite was true for 13-17 year old girls, where intentional injury rates were twice as high as unintentional injury rates. This difference was most notable among 16-17 year old girls where there were 11.5 per 10,000 intentional alcohol-related injuries in 2009 compared to 4.8 per 10,000 unintentional alcohol-related injuries (Figure 2). In England, the predominant cause of alcohol-related injury was intentional, with 16-17 year old girls in particular showing the highest rates (10.6 per 10,000 in 2009). This was one of the few trends that mirrored the pattern shown in Western Australia.

Alcohol-related injuries that were recorded as intentional are shown according to whether records indicated violence (which were predominantly assaults) or self-harm (Figure 3). In Western Australia, alcohol-related injuries caused by violence increased for both

For peer review only - http://bmjopen.bmj.com/site/about/guidelines.xhtml

For peer review only

genders. This is in contrast to England where violence related injury rates remained relatively stable. In WA violence-related injuries for boys aged 13-17 year old (4 per 10,000 in 2009) were the most prevalent intentional injuries, with the highest being for 16-17 year old boys at 8 per 10,000, increasing 4.8% (95%CI:2.8, 6.7, p<0.001) per year. For girls 16-17 year olds have higher rates (2.7 per 10,000 in 2009) compared to 13-15 year old girls, however they remained stable from 1990-2009 but increased significantly for 13-15 year olds by 7.1% per year (Figure 3). Self-harm predominated among girls in both WA and England, showing increasing rates in both countries, but a decline in England since 2007.

Unintentional alcohol-related injury rates have also increased in Western Australia for both boys and girls between 1990 and 2009 apart from 13-15 year old boys which remained stable. In England, unintentional alcohol-related injury rates have generally fallen or remained stable across both genders.

Absolute rates of alcohol-related injuries are shown for both countries for 2009, the most recent year in which comparable data was available for Western Australia (Table 1). For 13-17 year olds overall, Western Australia had higher rates of alcohol-related injuries in all categories other than self-harm. In both countries, 16-17 year olds had the highest rate of alcohol-related injuries, however the rate was twice as high among 16-17 year old boys in Western Australia (27.1/10,000) than in England (13.2/10,000). This pattern was consistent across both intentional and unintentional injuries. England had higher rates of alcohol-related self-harm, particularly among girls aged 13-15 (5.1/10,000) than Western Australia (2.5/10,000).

For peer review only - http://bmjopen.bmj.com/site/about/guidelines.xhtml

Table 1. Alcohol-related injury admission rates for children in Western Australia and England aged 13-17 years, 2009

Age group	Alcohol-related cause of injury	Western Australia			England		
		Boys<br>Absolute rate 2009<br>(per 10,000)	Girls<br>Absolute rate 2009<br>(per 10,000)	Total<br>Absolute rate 2009<br>(per 10,000)	Boys<br>Absolute rate 2009<br>(per 10,000)	Girls<br>Absolute rate 2009<br>(per 10,000)	Total<br>Absolute rate 2009<br>(per 10,000)
13-15 years	Injury	3.7 (2.3, 5.9)	6.0 (4.1, 8.8)	4.8 (3.6, 6.5)	4.6 (4.2, 5.1)	8.7 (8.1, 9.3)	6.6 (6.3, 7.0)
	Intentional	1.5 (0.7, 3.2)	3.7 (2.3, 6.0)	2.6 (1.7, 3.9)	1.8 (1.5, 2.1)	5.5 (5.0, 6.0)	3.6 (3.3, 3.9)
	Violence	0.9 (0.3, 2.3)	1.1 (0.5, 2.8)	1.0 (0.5, 1.9)	0.7 (0.5, 0.9)	0.5 (0.4, 0.7)	0.6 (0.5, 0.7)
	Self-harm	0.6 (0.2, 2.0)	2.5 (1.4, 4.6)	1.6 (0.9, 2.6)	1.1 (1.0, 1.4)	5.1 (4.7, 5.6)	3.1 (2.8, 3.3)
	Unintentional	2.2 (1.2, 4.0)	1.6 (0.8, 3.4)	1.9 (1.2, 3.0)	2.8 (2.5, 3.2)	3.6 (3.2, 4.0)	3.2 (2.9, 3.5)
16-17 years	Injury	27.1 (21.9, 33.5)	16.6 (12.6, 22.0)	22.0 (18.6, 26.1)	13.2 (12.4, 14.1)	14.1 (13.2, 15.0)	13.6 (13.0, 14.3)
	Intentional	12.9 (9.5, 17.6)	11.5 (8.3, 16.2)	12.2 (9.7, 15.4)	6.9 (6.3, 7.6)	10.6 (9.8, 11.5)	8.7 (8.2, 9.2)
	Violence	8.4 (5.7, 12.3)	2.7 (1.4, 5.4)	5.6 (4.0, 7.9)	2.9 (2.5, 3.3)	0.5 (0.4, 0.7)	1.7 (1.5, 2.0)
	Self-harm	4.8 (2.9, 8.0)	8.8 (6.0, 13.0)	6.8 (5.0, 9.2)	4.1 (3.7, 4.6)	10.2 (9.4, 11.0)	7.1 (6.6, 7.5)
	Unintentional	12.9 (9.5, 17.6)	4.8 (2.8, 8.0)	8.9 (6.8, 11.7)	6.5 (5.9, 7.1)	3.9 (3.4, 4.4)	5.2 (4.8, 5.6)

For peer review only - http://bmjopen.bmj.com/site/about/guidelines.xhtml

For peer review only

13-17 years	Injury	13.1 (10.8, 15.9)	10.3 (8.2, 12.9)	11.7 (10.1, 13.6)	8.2 (7.8, 8.7)	10.9 (10.4, 11.5)	9.5 (9.2, 9.9)
	Intentional	6.1 (4.6, 8.1)	6.9 (5.2, 9.0)	6.5 (5.3, 7.9)	3.9 (3.6, 4.2)	7.6 (7.2, 8.0)	5.7 (5.4, 6.0)
	Violence	3.9 (2.7, 5.6)	1.8 (1.0, 3.1)	2.9 (2.1, 3.9)	1.6 (1.4, 1.8)	0.5 (0.4, 0.6)	1.1 (1.0, 1.2)
	Self-harm	2.3 (1.5, 3.7)	5.1 (3.7, 7.0)	3.7 (2.8, 4.8)	2.4 (2.2, 2.6)	7.2 (6.8, 7.7)	4.7 (4.5, 5.0)
	Unintentional	6.5 (4.9, 8.5)	2.9 (1.9, 4.4)	4.7 (3.7, 6.0)	4.3 (4.0, 4.7)	3.7 (3.4, 4.0)	4.0 (3.8, 4.2)

For peer review only

Further analysis of the alcohol-related injury admission was conducted for Western Australia. Overall, the average length of stay associated with alcohol-related admissions was 2.6 days. We found that of self-harm injuries in boys, 24% were alcohol-related, compared to 13% of self-harm injuries in girls. For both genders, 15-17% of assault injuries were alcohol-related, compared to only 3% of accidental injuries. For 16-17 year olds alcohol was associated with 18% of assault-related injuries compared to 12% in 13-15 year olds. In 2009 this was even higher at 27% of assault-related injuries being associated with alcohol in the 16-17 year old age group. The predominant type of alcohol-related assault was assault by bodily force at (53%), followed by assault by unspecified means (18%), assault by blunt object (14%) and assault by sharp object (10%). In 3% of alcohol-related assault admissions the assault was rape.

DISCUSSION

Despite the decline in hospital admissions in Western Australia from 1990 to 2009 for injury for adolescents aged 13-17 there has been an increase in alcohol-related injuries by 2.3% per year. This increase has been largest in the 16-17 year old age group with boys aged 16-17 years having the highest rates. However girls in both the 13-15 and 16-17 age groups had the greatest increase in trends of 2.7% and 3.2% per year respectively. This substantiates and builds on a previous Australian study showing increases in alcohol-related injury admissions in 16-17 year olds till 2005 and a large rise in hospital presentations for girls.[4]

For peer review only - http://bmjopen.bmj.com/site/about/guidelines.xhtml

In contrast to the increasing rates of alcohol-related injuries in Western Australia, England's rates remained relatively stable over the study period. Most age and gender groups have shown decreasing or stable rates of alcohol-related injuries since 2007, with the exception of 16-17 year old girls. This suggests that alcohol-related harm in adolescents is a problem that can be reduced. Although coding practices can affect the magnitude of the trend, a decrease in overall alcohol-related harms was found despite an increase in England's use of secondary diagnosis codes[18], and is consistent with surveys of 11-15 year olds from 2001-2011 showing a decrease in frequent drinking.[19] In addition, England's Health Behaviour in School-aged Children Report found from 2002-2014 there was a 23-26% reduction in the proportion of 15 year olds reporting drinking to excess.[20] Recent data from Public Health England has also found a 13.9% fall in hospital admissions caused by alcohol in England for under 18's which corroborates our findings.[21]

In England, self-harm was the leading cause of alcohol-related injury particularly among girls, an issue which warrants further attention. Self-harm was also the most common form of intentional alcohol-related injury for girls in Western Australia. Research has found many adolescents admitted for injuries with self-harm also had drug or alcohol misuse coded and increased likelihood of having experienced violence, suggesting a need for strategies that address multiple forms of adversity. [22]

A large minority of injuries in Western Australia were unintentional injuries however there was a rise in violence-related admissions. Overall alcohol was associated with 16% of assault-related admissions and this was higher in 2009 for adolescents aged 16-17

For peer review only - http://bmjopen.bmj.com/site/about/guidelines.xhtml

years, with 20% of assaults associated with alcohol for girls and 30% for boys. This highlights the growing issue in Australia with the rise in violent injuries related to alcohol and the associated harm that adolescents are experiencing. It is important that this issue be monitored, firstly so we know the extent of harm and the burden on the health system of alcohol-related injuries and secondly that as strategies are implemented to reduce this burden we can look at the impact on the rate of admissions.

Unfortunately our data only captures when the individual admitted for the injury has an alcohol-related diagnosis not when the perpetrator had been affected by alcohol. Therefore this would certainly underestimate the rates of injury associated with alcohol use which is also important in capturing the burden. Research suggests alcohol use by the perpetrator was a factor in over half of physical and sexual assaults,[23] and has established associations with child maltreatment [24 25] and road traffic crashes.[26] Future research could use data from police and corrective services to investigate alcohol-related harm. Also our data only relates to injuries requiring hospitalisation not those seen in the Emergency Departments (ED) therefore our injury admissions would relate to more serious injuries and violence. Another limitation is that changes in coding over time may influence apparent trends. Our study shows that at least in WA there is an increase in recognition (whether due to more rigorous reporting or as a rise in actual incidents) of the influence of alcohol in injury admissions.

Using hospital admissions data allows monitoring of trends over time and country comparisons can generate hypotheses about policies or service factors that may account for differences. This is the first study examining and comparing trends in alcohol-related

For peer review only - http://bmjopen.bmj.com/site/about/guidelines.xhtml

harm in adolescents in Western Australia and England. Identification of age and gender groups at greatest risk, and the types of harm they experience is necessary to inform public health initiatives targeting alcohol-related harm in adolescents. In England it is unclear which specific policies or practices have influenced the decline, but a strategy for reducing alcohol-related harm has been in place since 2004. An annual tax escalator was implemented to boost duties on all alcohol[27] in 2008 and Challenge 25 was subsequently introduced, requiring identification from people who look less 25 years old when purchasing alcohol. Researchers have also stated that the economic downturn in England and the rise in alcohol taxation may have contributed to stemming the rise in alcohol-related deaths[28]. English researchers have also noted falls in other adversity-related admissions from 2005-11 and discussed the implementation of programs (eg. Sure Start, Alcohol Misuse Enforcement Campaign) which may have influenced rates[29].

Some strategies and programs are in place in Australia to address youth drinking and alcohol-related harm.[2 3] These appear to be having some positive effects, with adolescents being the only group that has shown a decrease in the proportion of people who drink at risky levels.[3] Nonetheless, our research shows that rates of alcohol-related injury admissions increased in Western Australia, consistent with findings for Victoria, Australia.[4] Possible explanations for this discordance include the hypothesis that sub-groups excluded from surveys (such as homeless or truant youth, those who left school prior to Year 12 for school surveys, or those that chose not to participate) have had increased drinking or that sub-groups have increased excessive and risky drinking.[30]

Australia and England follow a number of the World Health Organisation recommendations for reducing alcohol-related harm including maximum blood alcohol

For peer review only - http://bmjopen.bmj.com/site/about/guidelines.xhtml

levels while driving, and restrictions on age, sales to intoxicated people, and promotion of alcoholic beverages at activities targeting young people.[11] However more clearly needs to be done to reduce the rising rates of alcohol-related harm in Western Australia and in England despite the declining rates Professor Kevin Fenton, Director of Health and Wellbeing at Public Health England has stated that 'while this is promising… current levels of harm caused by alcohol remain unacceptably high, especially within the most deprived communities, who suffer the most from poor health in general.'[31]

Evidence suggests the optimal package of interventions also includes measures to reduce the affordability and availability of alcohol.[32-34] There is evidence that pricing policy can significantly reduce alcohol consumption [35 36] and alcohol-related hospital admissions [34] and deaths [37]. Minimum unit pricing or volumetric pricing are considered effective and cost-effective solutions.[38] In 2012, Scotland followed Canada in legislating for minimum alcohol unit pricing.[39] Recent research has found the majority of young risky drinkers support evidence-based policies including price increases if revenue is used to support treatment and prevention of alcohol problems.[40]

For peer review only

For peer review only - http://bmjopen.bmj.com/site/about/guidelines.xhtml

CONTRIBUTORSHIP STATEMENT

O'Donnell conceptualised the paper, developed the statistical plan, wrote a preliminary draft and revised the paper. Gilbert contributed to the conceptualisation of the paper, contributed to the draft and revised the paper. Sims cleaned and analysed the Western Australian data, contributed to the draft and revised the paper. Gonzalez-Izquierdo cleaned and analysed England's data, contributed to the draft and revised the paper. Maclean contributed to the interpretation of findings, the drafting of the paper and revised the paper. Stanley contributed to the conceptualisation of the paper and revised the paper.

COMPETING INTERESTS

The authors have no competing interests.

FUNDING AND ACKNOWLEDGEMENTS

The Policy Research Unit in the Health of Children, Young People and Families (CPRU), which is funded by the England Department of Health Policy Research Programme. This is an independent report commissioned and funded by the Department of Health. We thank members of CPRU: Terence Stephenson, Catherine Law, Amanda Edwards, Steve Morris, Helen Roberts, Cathy Street, Russell Viner and Miranda Wolpert. RG and AGI were also partly supported by awards establishing the Farr Institute of Health Informatics Research (MR/K006584/1). Melissa O'Donnell was supported by a National Health and Medical Research Council Early Career Fellowship [1012439]. This research was also supported by an Australian Research Council Linkage Project Grant [LP100200507] and an Australian Research Council Discovery Grant [DP110100967]. We also acknowledge the partnership of the Western Australian Government Departments of Health, Child Protection, Education, Disability Services, Corrective Services and the Attorney General,

For peer review only - http://bmjopen.bmj.com/site/about/guidelines.xhtml

which provided data for this project. The views expressed are not necessarily those of the Government Departments that have commissioned or supported this project.

DATA SHARING STATEMENT

The data utilised in this paper is owned by our respective government departments and therefore would require permissions by these departments for others to access.

For peer review only

REFERENCES

1. Degenhardt L, Stockings E, Patton G, et al. The increasing global health priority of substance use in young people. The Lancet Psychiatry 2016;**3**(3):251-64.
2. Moodie R, Daube M, Carnell K, et al. Australia: The healthiest country by 2020 National Preventative Health Strategy - the roadmap for action. Canberra Australian Government Preventative Health Taskforce, 2009.
3. Moodie R, Daube M, Zimmet P, et al. Taking Preventative Action: A Response to Australia: The Healthiest Country by 2020 - The Report of the National Preventative Health Task Force. Canberra: Australian Government Preventative Health Taskforce, 2010.
4. Livingston M. Recent trends in risky alcohol consumption and related harm among young people in Victoria, Australia. Australian and New Zealand Journal of Public Health 2008;**32**(3):266-71.
5. Morgan A, McAtamney A. Key issues in alcohol-related violence. Research in Practice: Summary no. 4. . Canberra: Australian Institute of Criminology, 2009.
6. Plant M, Plant M, Thornton C. People and places: Some factors in the alcohol-violence link. Journal of Substance Use 2002;**7**(4):207-13.
7. Krug EG, Dahlberg LL, Mercy JA, et al. World report on violence and health. Geneva: World Health Organization, 2002.
8. World Health Organisation. Global strategy to reduce the harmful use of alcohol. Geneva: WHO, 2010.
9. Morgan A, McAtamney A. Key issues in alcohol-related violence. Research in Practice: Summary no 4. Canberra: Australian Institute of Criminology, 2009.
10. Gonzalez-Izquierdo A, Ward A, O'Donnell M, et al. Cross-country comparison of victimisation-related injury admission in children and adolescents in England and Western Australia. BMC Health Services Research 2013;**13**(1):260.
11. WHO. Global status report on alcohol and health 2014 Geneva: World Health Organization, 2014.
12. Toumbourou JW, Stockwell T, Neighbors C, et al. Adolescent Health 4: Interventions to reduce harm associated with adolescent substance use. Lancet 2007;**369**(9570):1391-401.
13. Egerton-Warburton D, Gosbell A, Wadsworth A, et al. Survey of alcohol-related presentations to Australasian emergency departments. Medical Journal of Australia 2014;**201**(10):584-87.
14. Gonzalez-Izquierdo A, Ward A, O'Donnell M, et al. Cross-country comparison of victimisation-related injury admission in children and adolescents in England and Western Australia. BMC Health Services Research 2013;**13**:260.
15. NHS. The information centre for health and social care. HES online. The approvals process. 2012. 6-8-2012.
16. Kuhn L, Davidson LL, Durkin MS. Use of poisson regression and time series analysis for detecting changes over time in rates of child injury following a prevention program. American Journal of Epidemiology 1994;**140**(10):943-55.
17. SAS Version 9.1 [program]. Cary NC: SAS Institute Inc., 2004.
18. Lifestyle Statistics Health and Social Care Information Centre. Statistics on Alcohol: England, 2013. England: UK Government, 2013.
19. Fuller E, Gill V, Hawkins V, et al. Smoking, drinking and drug use among young people in England in 2011, NatCen Social Research. London, 2012.

For peer review only - http://bmjopen.bmj.com/site/about/guidelines.xhtml

20. Brooks F, Magnusson J, Klemera E, et al. HBSC England National Report 2014. Hatfield, UK: University of Hertfordshire, 2015.
21. Public Health England. Local Alcohol Profiles for England, June 2015. Statistical Bulletin. London: Public Health England, 2015.
22. Herbert A, Gilbert R, González-Izquierdo A, et al. Violence, self-harm and drug or alcohol misuse in adolescents admitted to hospitals in England for injury: a retrospective cohort study. BMJ Open 2015;**5**(2):e006079.
23. Connor J, You R, Casswell S. Alcohol-related harm to others: a survey of physical and sexual assault in New Zealand. New Zealand Medical Journal 2009;**122**(1303).
24. Gilbert R, Widom CS, Browne K, et al. Burden and consequences of child maltreatment in high-income countries. Lancet 2009;**373**(9657):68-81.
25. Laslett A-M, Ferris J, Dietze P, et al. Social demography of alcohol-related harm to children in Australia. Addiction 2012;**107**(6):1082-89.
26. Bramwell J, Hill D, Thompson PE. Reported road crashes in Western Australia 2012. Perth: Road Safety Council of Western Australia, 2014.
27. Gilmore I, Anderson W, Bauld L, et al. Health first: An evidence-based alcohol strategy for the UK Stirling: University of Stirling, 2013.
28. Sheron N, Gilmore I. Effect of policy, econmics, and the changing alcohol marketplace on alcohol related deaths in England and Wales. British Medical Journal 2016;**353**:i1860
29. Herbert A, Gonzalez-Izquierdo A, McGhee J, et al. Time-trends in rates of hospital admissions of adolescents for violent, self-inflicted or drug/alcohol-relted injury in England and Scotland, 2005-11: population-based analysis. Journal of Public Health 2016;doi:10.1093/pubmed/fdw020.
30. Livingston M, Matthews S, Barratt MJ, et al. Diverging trends in alcohol consumption and alcohol-related harm in Victoria. Australian and New Zealand Journal of Public Health 2010;**34**(4):368-73.
31. Public Health England. Alcohol-related hospital admissions decrease among under 18s. London: Public Health England, 2015.
32. Anderson P, Chisholm D, Fuhr DC. Alcohol and global health 2: Effectiveness and cost-effectiveness of policies and programmes to reduce the harm caused by alcohol. Lancet 2009;**373**(9682):2234-46.
33. Casswell S, Thamarangsi T. Alcohol and global health 3: Reducing harm from alcohol: call to action. Lancet 2009;**373**(9682):2247-57.
34. Stockwell T, Zhao J, Martin G, et al. Minimum alcohol prices and outlet densities in British Columbia, Canada: Estimated impacts on alcohol-attributable hospital admissions. American Journal of Public Health 2013;**103**(11):2014-20.
35. Wagenaar AC, Salois MJ, Komro KA. Effects of beverage alcohol price and tax levels on drinking: a meta-analysis of 1003 estimates from 112 studies. Addiction 2009;**104**(2):179-90.
36. Stockwell T, Zhao J, Giesbrecht N, et al. The raising of minimum alcohol prices in Saskatchewan, Canada: Impacts on consumption and implications for public health. American Journal of Public Health 2012;**102**(12):e103-e10.
37. Chikritzhs T, Stockwell T, Pascal R. The impact of the Northern Territory's Living With Alcohol program, 1992–2002: revisiting the evaluation. Addiction 2005;**100**(11):1625-36.

For peer review only - http://bmjopen.bmj.com/site/about/guidelines.xhtml

38. Sharma A, Vandenberg B, Hollingsworth B. Minimum pricing of alcohol versus volumetric taxation: Which policy will reduce heavy consumption without adversely affecting light and moderate consumers? PLoS ONE 2014;**9**(1):1-13.
39. Leicester A. Alcohol pricing and taxation policies, IFS Briefing Note BN124: Institute for Fiscal Studies, 2011.
40. Lam T, Lenton S, Burns L, et al. Alcohol policy impact on young risky drinkers and their support for proposed measures. Australian and New Zealand Journal of Public Health 2015;**39**(2):12-134.

For peer review only

For peer review only - http://bmjopen.bmj.com/site/about/guidelines.xhtml

Figure 1. Rate of hospital admissions for alcohol-related injury in Western Australia and England (per 10,000 persons) for adolescents, aged 13-15 and 16-17 years. Light solid= Alcohol-related injury (13-15yrs); dark solid=Alcohol-related injury (16-17yrs)

See Supplementary file for Figure 1

Figure II. Rate of intentional and unintentional alcohol-related injury hospital admissions in Western Australia and England (per 10,000 persons) for adolescents aged 13-15 and 16-17years. Light dash=intentional (13-15yrs); Light solid=unintentional (13-15yrs); dark dash=intentional (16-17yrs); dark solid=unintentional (16-17yrs).

See Supplementary file for Figure 2

Figure III. Rate of intentional alcohol-related injury hospital admissions in Western Australia and England (per 10,000 persons) for adolescents aged 13-17 years, by violence-related and self-harm admissions. Dark dash=violence related (13-17yrs); dark solid=self-harm (13-17yrs).

See Supplementary file for Figure 3.

For peer review only

For peer review only - http://bmjopen.bmj.com/site/about/guidelines.xhtml

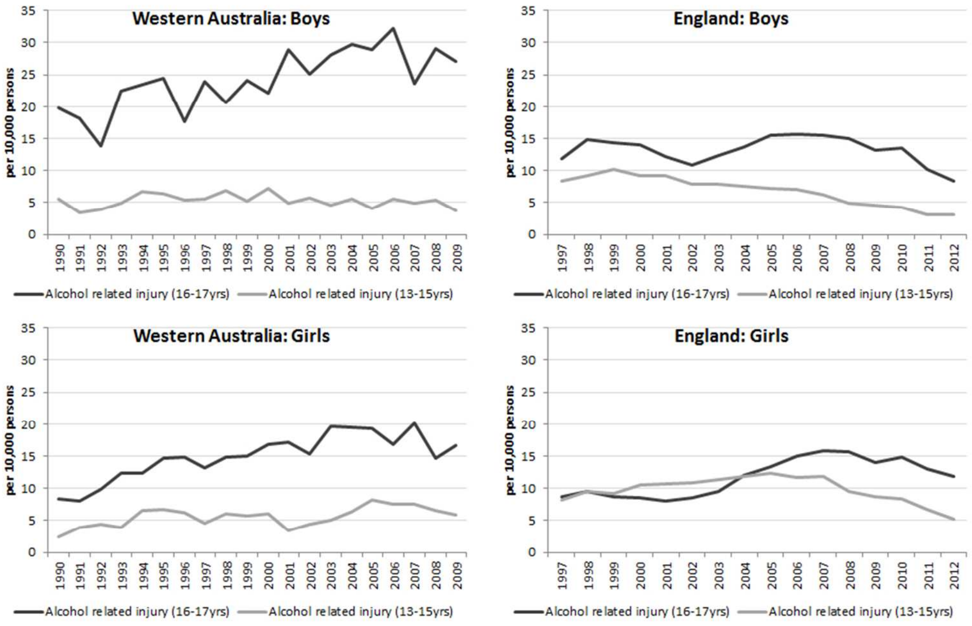

Figure 1. Rate of hospital admissions for alcohol-related injury in Western Australia and England (per 10,000 persons) for adolescents, aged 13-15 and 16-17 years. Light solid= Alcohol-related injury (13-15yrs); dark solid=Alcohol-related injury (16-17yrs)

80x51mm (300 x 300 DPI)

view only

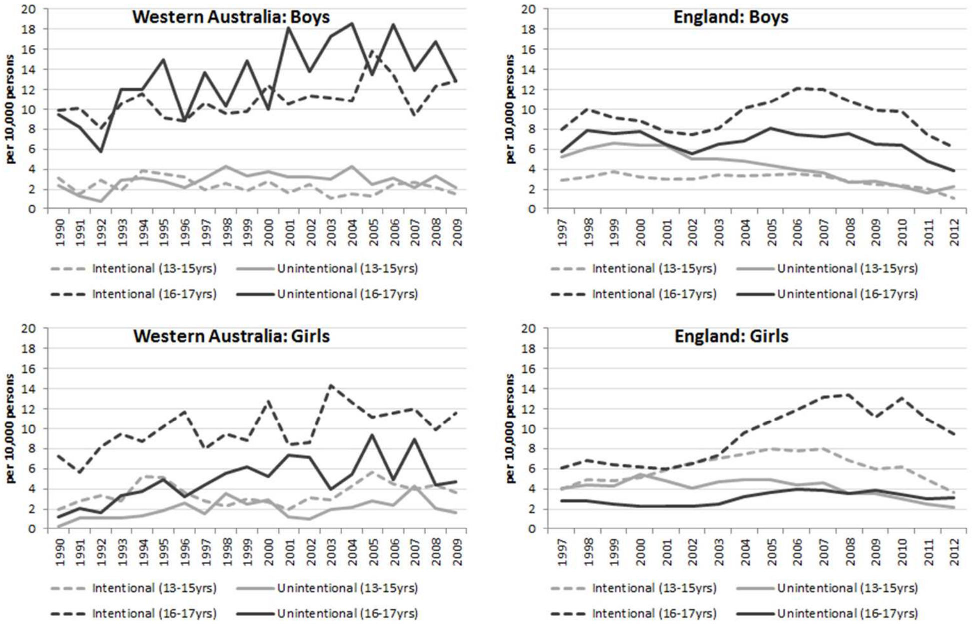

Figure II. Rate of intentional and unintentional alcohol-related injury hospital admissions in Western Australia and England (per 10,000 persons) for adolescents aged 13-15 and 16-17years. Light dash=intentional (13-15yrs); Light solid=unintentional (13-15yrs); dark dash=intentional (16-17yrs); dark solid=unintentional (16-17yrs).

80x51mm (300 x 300 DPI)

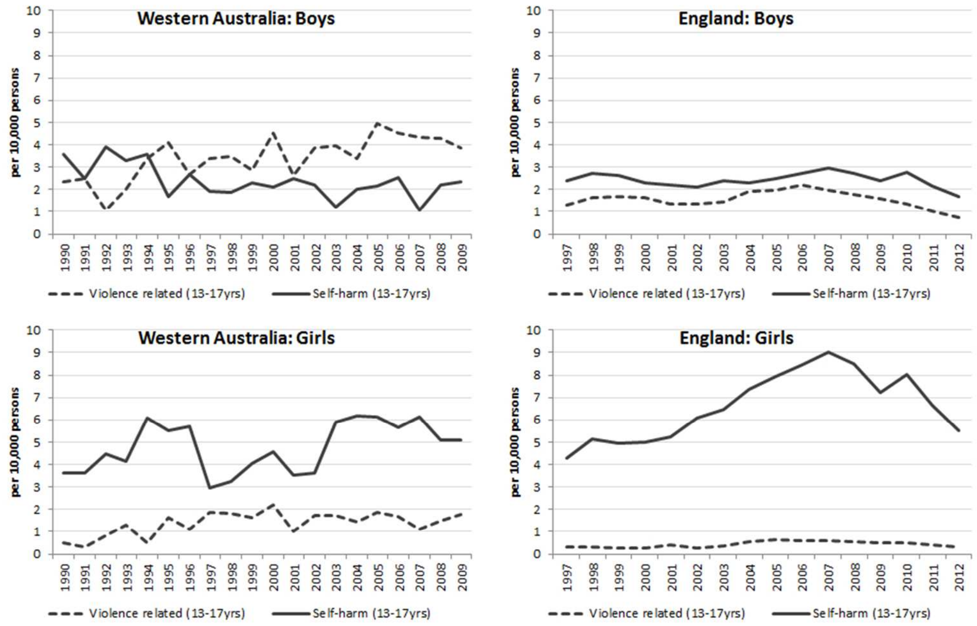

Figure III. Rate of intentional alcohol-related injury hospital admissions in Western Australia and England (per 10,000 persons) for adolescents aged 13-17 years, by violence-related and self-harm admissions. Dark dash=violence related (13-17yrs); dark solid=self-harm (13-17yrs).

80x51mm (300 x 300 DPI)

STROBE Statement—checklist of items that should be included in reports of observational studies

	Item No	Recommendation
**Title and abstract**	A - Title<br>B - Abstract	(*a*) Indicate the study's design with a commonly used term in the title or the abstract
		(*b*) Provide in the abstract an informative and balanced summary of what was done and what was found
**Introduction**		
Background/rationale	Pg 5 and 6	Explain the scientific background and rationale for the investigation being reported
Objectives	Pg 6	State specific objectives, including any prespecified hypotheses
**Methods**		
Study design	Pg 6 and 8	Present key elements of study design early in the paper
Setting	Pg 6	Describe the setting, locations, and relevant dates, including periods of recruitment, exposure, follow-up, and data collection
Participants	A- Pg6 and 7 population based cohort so not necessary to discuss follow-up	(*a*) *Cohort study*—Give the eligibility criteria, and the sources and methods of selection of participants. Describe methods of follow-up<br>*Case-control study*—Give the eligibility criteria, and the sources and methods of case ascertainment and control selection. Give the rationale for the choice of cases and controls<br>*Cross-sectional study*—Give the eligibility criteria, and the sources and methods of selection of participants
		(*b*) *Cohort study*—For matched studies, give matching criteria and number of exposed and unexposed<br>*Case-control study*—For matched studies, give matching criteria and the number of controls per case
Variables	Pg 6 and 7	Clearly define all outcomes, exposures, predictors, potential confounders, and effect modifiers. Give diagnostic criteria, if applicable
Data sources/ measurement	Pg 6 and 7	For each variable of interest, give sources of data and details of methods of assessment (measurement). Describe comparability of assessment methods if there is more than one group
Bias	None (discussed in limitations – eg coding changes)	Describe any efforts to address potential sources of bias
Study size	Not Applicable Population based cohort study	Explain how the study size was arrived at
Quantitative variables	Pg 8	Explain how quantitative variables were handled in the analyses. If applicable, describe which groupings were chosen and why
Statistical methods	A and B – Pg 8<br>C – None<br>D- Not applicable<br>E - None	(*a*) Describe all statistical methods, including those used to control for confounding
		(*b*) Describe any methods used to examine subgroups and interactions
		(*c*) Explain how missing data were addressed
		(*d*) *Cohort study*—If applicable, explain how loss to follow-up was addressed<br>*Case-control study*—If applicable, explain how matching of cases and controls was addressed

For peer review only - http://bmjopen.bmj.com/site/about/guidelines.xhtml

Cross-sectional study—If applicable, describe analytical methods taking account of sampling strategy	
(*e*) Describe any sensitivity analyses	

For peer review only

For peer review only - http://bmjopen.bmj.com/site/about/guidelines.xhtml

**Results**		
Participants	Pg 6- Population based cohort study	(a) Report numbers of individuals at each stage of study—eg numbers potentially eligible, examined for eligibility, confirmed eligible, included in the study, completing follow-up, and analysed
		(b) Give reasons for non-participation at each stage
		(c) Consider use of a flow diagram
Descriptive data	A - Pg 6<br>B – Not applicable<br>C – Not applicable	(a) Give characteristics of study participants (eg demographic, clinical, social) and information on exposures and potential confounders
		(b) Indicate number of participants with missing data for each variable of interest
		(c) *Cohort study*—Summarise follow-up time (eg, average and total amount)
Outcome data	P9-15	*Cohort study*—Report numbers of outcome events or summary measures over time
		Case-control study—Report numbers in each exposure category, or summary measures of exposure
		Cross-sectional study—Report numbers of outcome events or summary measures
Main results	Pg 9-15	(*a*) Give unadjusted estimates and, if applicable, confounder-adjusted estimates and their precision (eg, 95% confidence interval). Make clear which confounders were adjusted for and why they were included
		(*b*) Report category boundaries when continuous variables were categorized
		(*c*) If relevant, consider translating estimates of relative risk into absolute risk for a meaningful time period
Other analyses	None	Report other analyses done—eg analyses of subgroups and interactions, and sensitivity analyses
**Discussion**		
Key results	Pg 15-16	Summarise key results with reference to study objectives
Limitations	Pg 17	Discuss limitations of the study, taking into account sources of potential bias or imprecision. Discuss both direction and magnitude of any potential bias
Interpretation	Pg 15-19	Give a cautious overall interpretation of results considering objectives, limitations, multiplicity of analyses, results from similar studies, and other relevant evidence
Generalisability	Pg 17	Discuss the generalisability (external validity) of the study results
**Other information**		
Funding	Pg 20	Give the source of funding and the role of the funders for the present study and, if applicable, for the original study on which the present article is based

*Give information separately for cases and controls in case-control studies and, if applicable, for exposed and unexposed groups in cohort and cross-sectional studies.

Note: An Explanation and Elaboration article discusses each checklist item and gives methodological background and published examples of transparent reporting. The STROBE checklist is best used in conjunction with this article (freely available on the Web sites of PLoS Medicine at http://www.plosmedicine.org/, Annals of Internal Medicine at http://www.annals.org/, and Epidemiology at http://www.epidem.com/). Information on the STROBE Initiative is available at www.strobe-statement.org.

For peer review only
